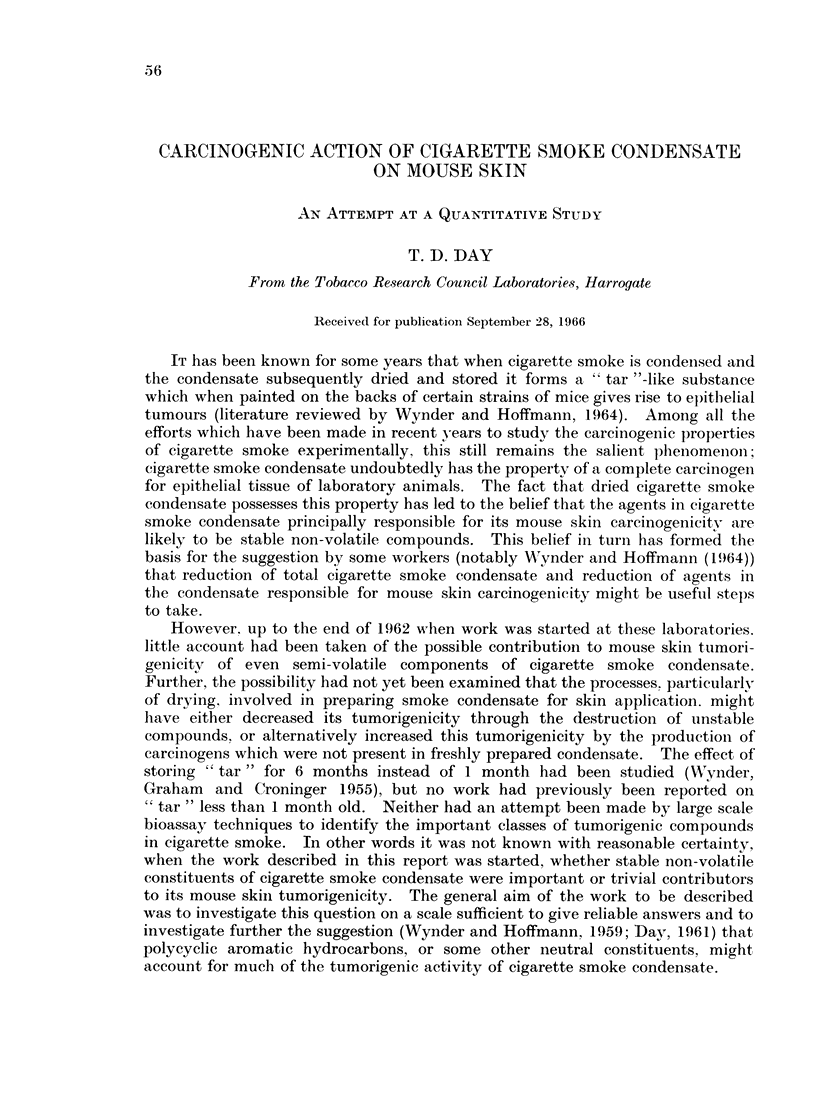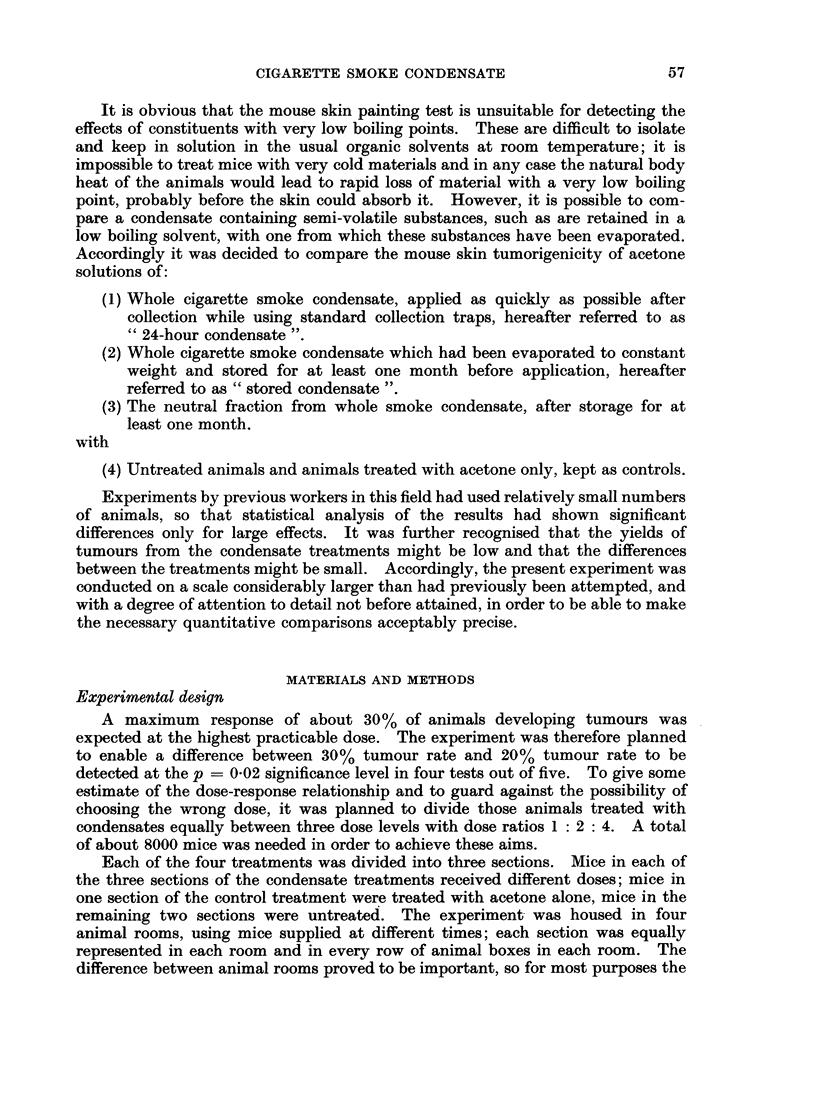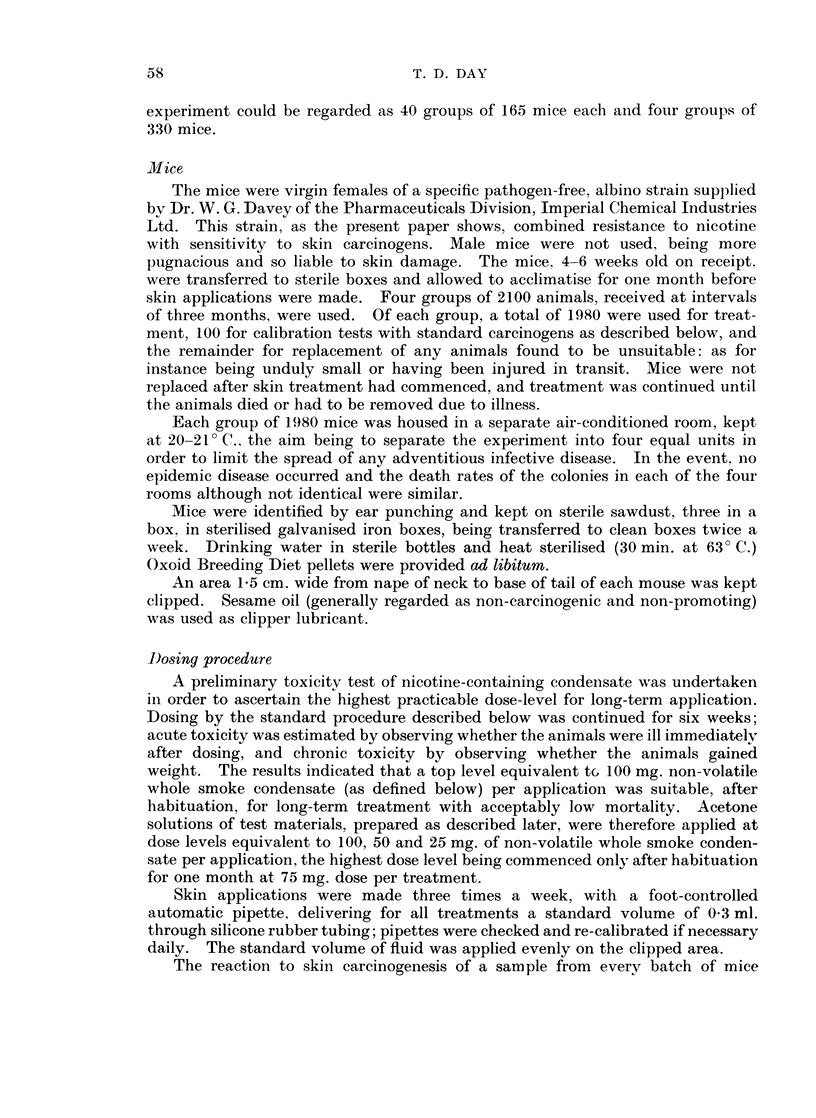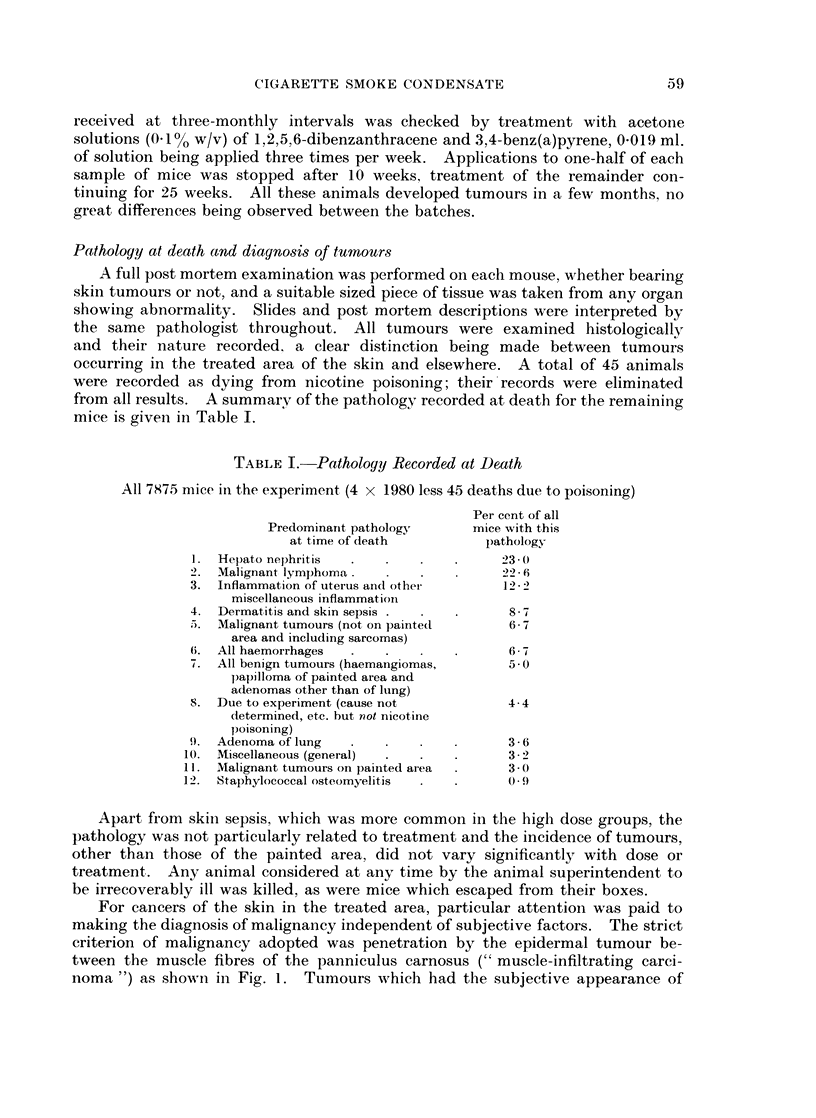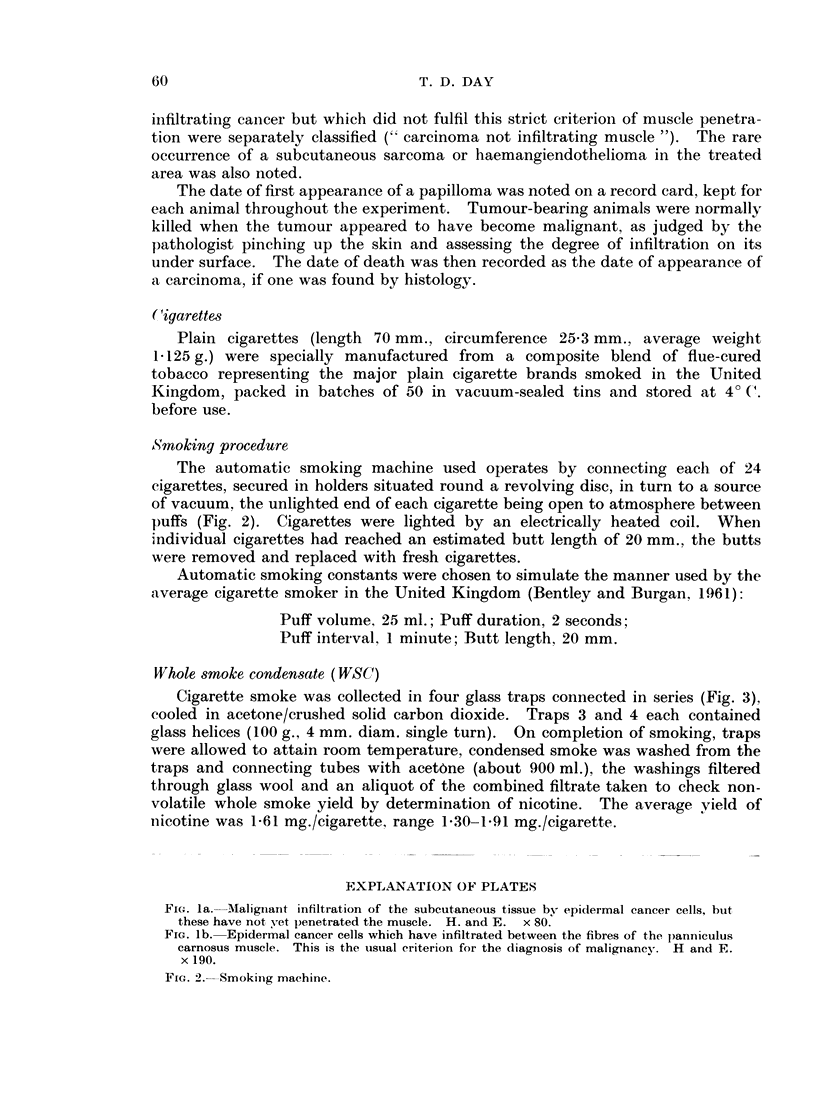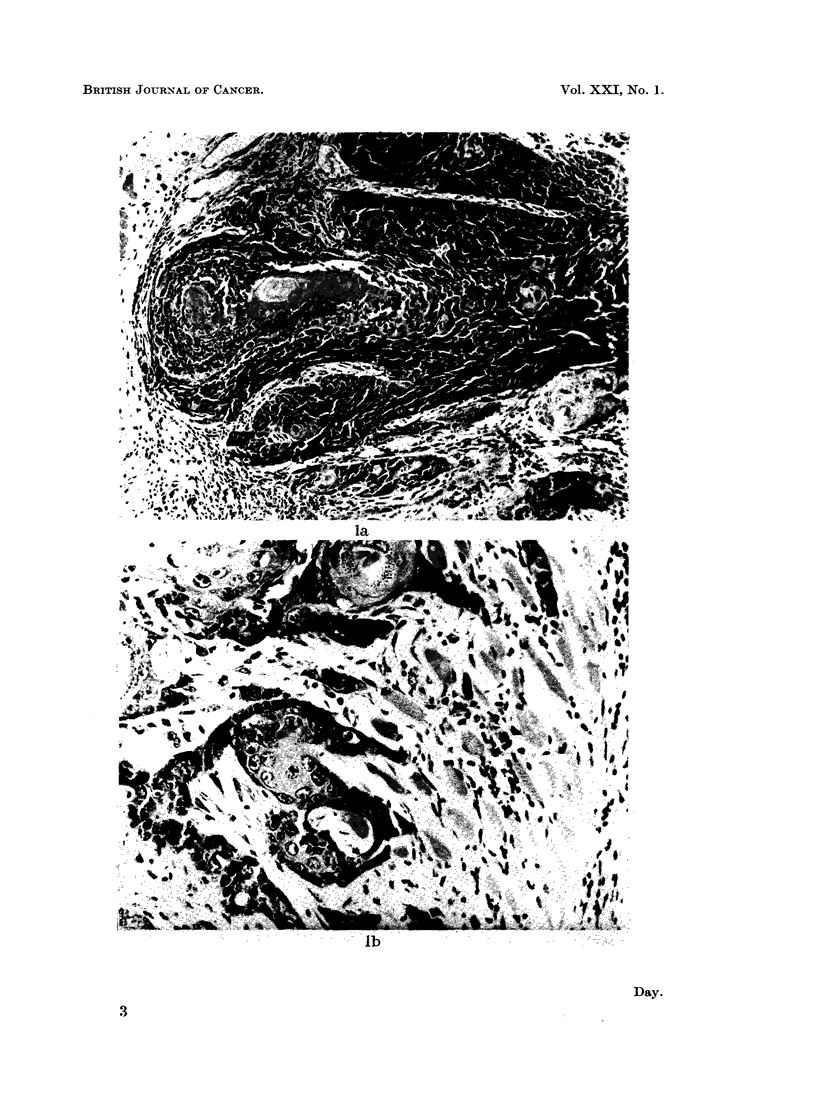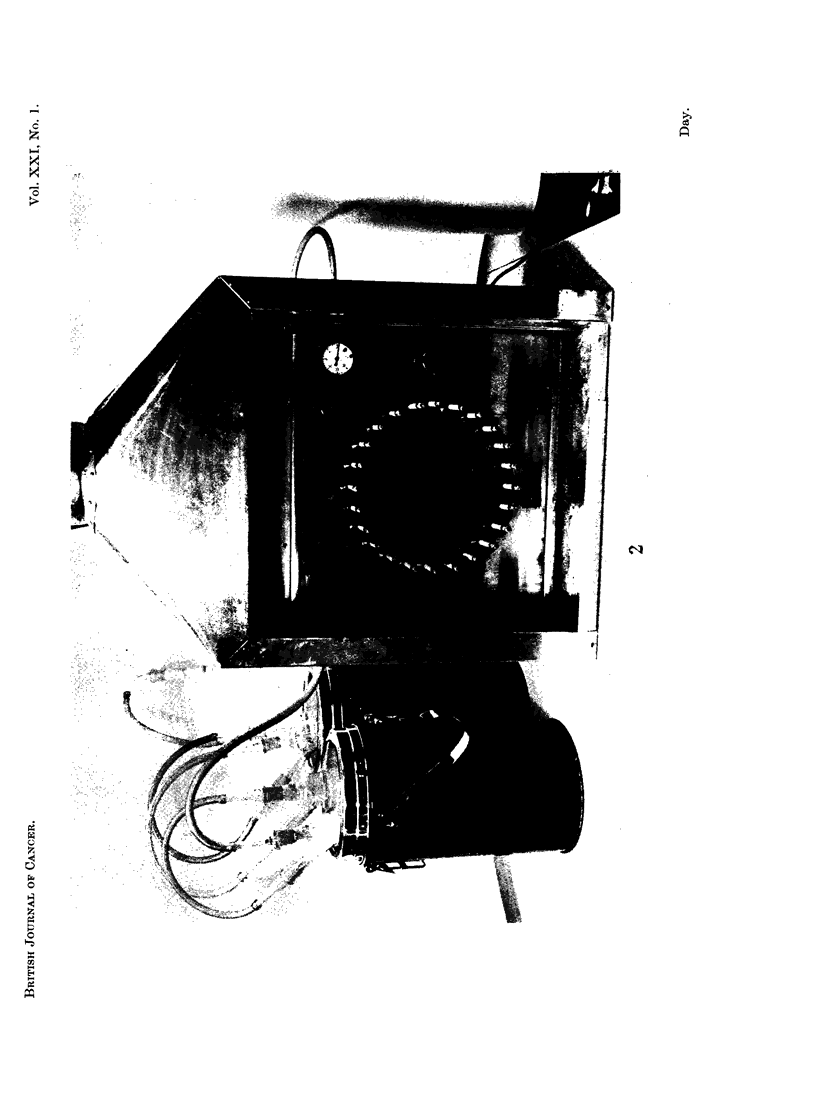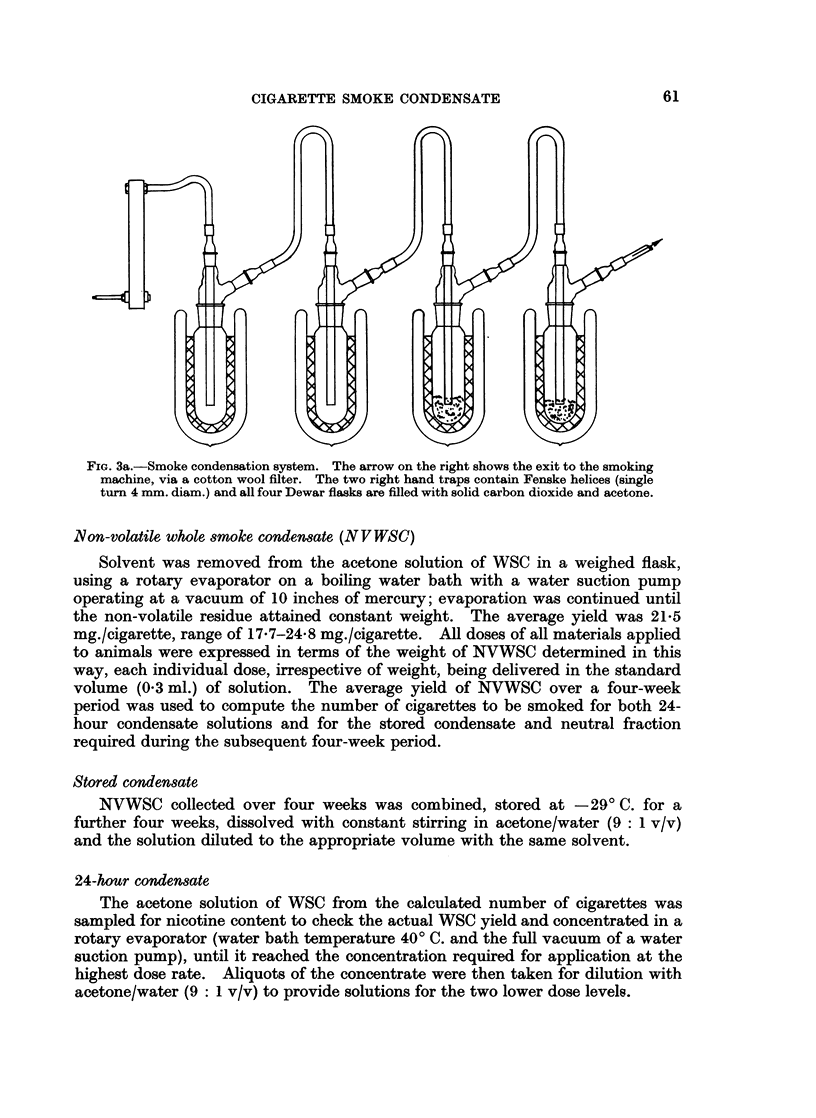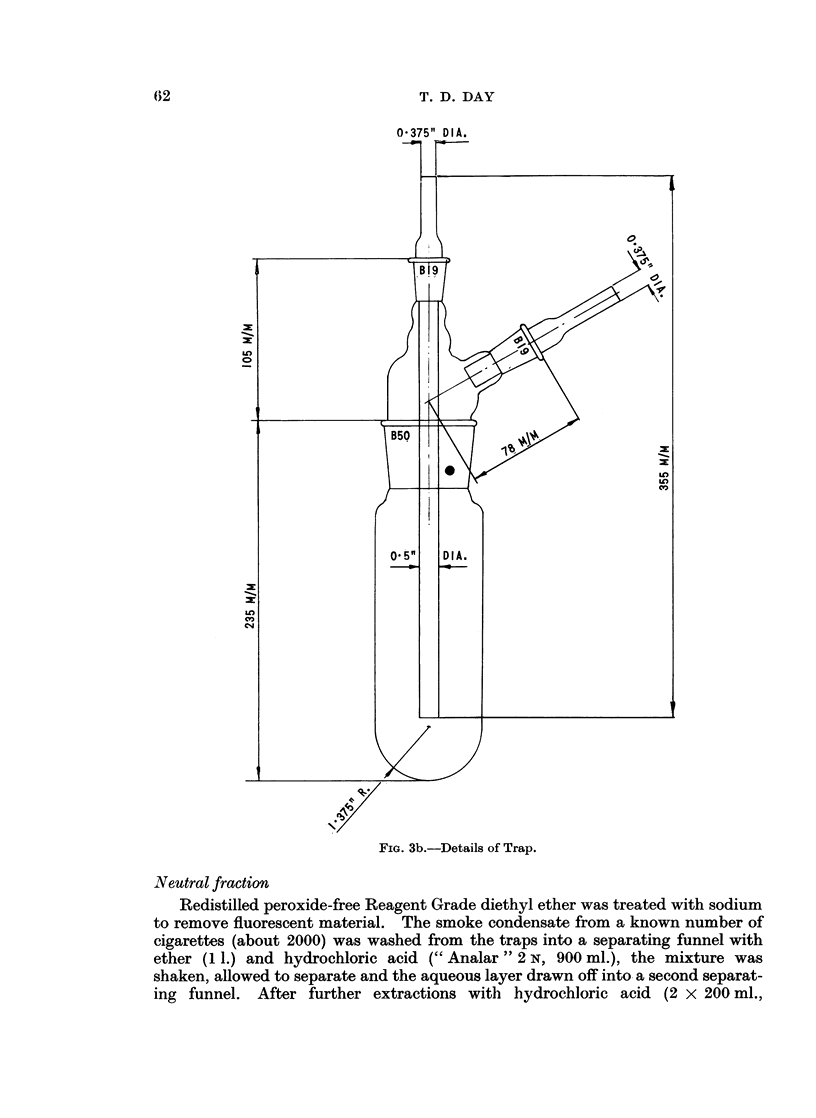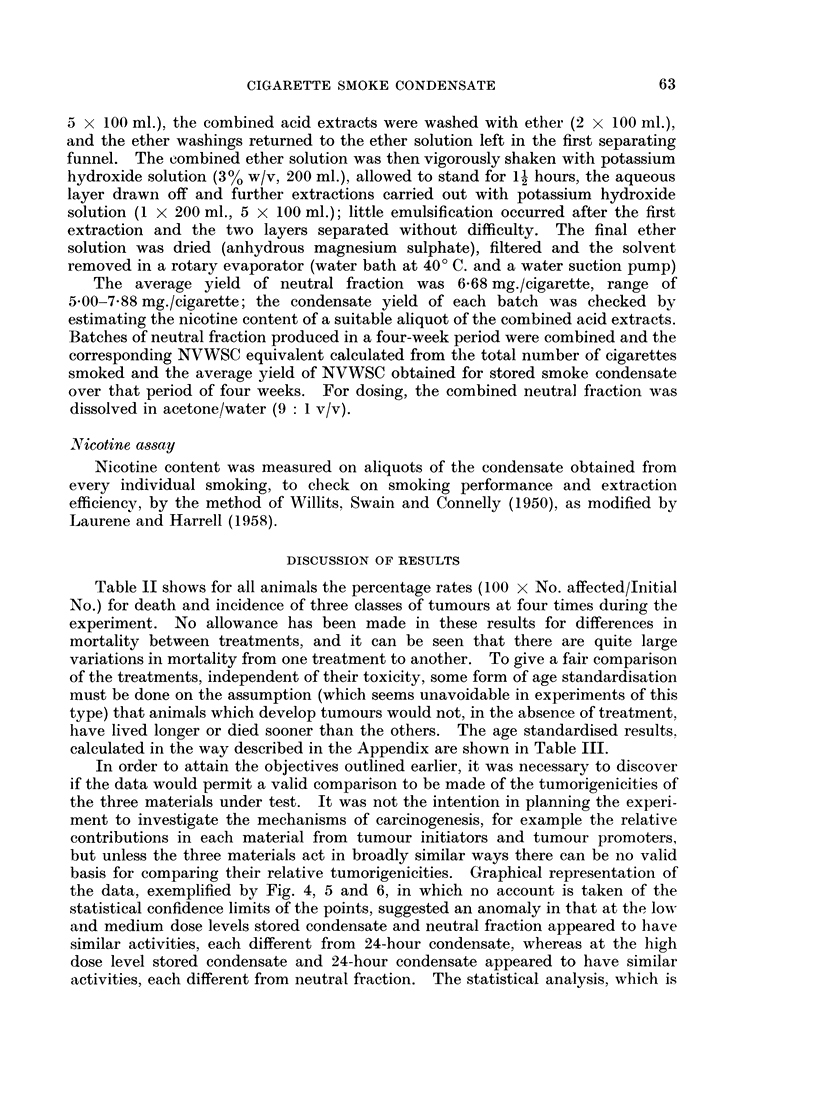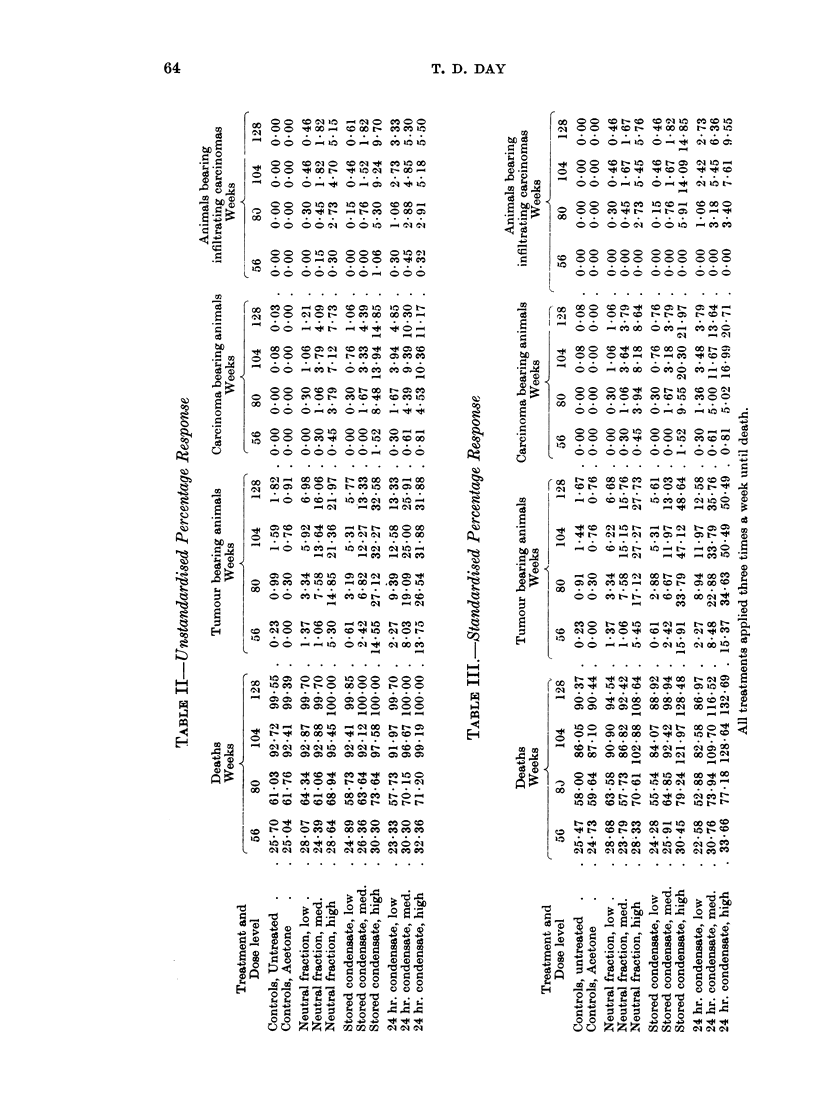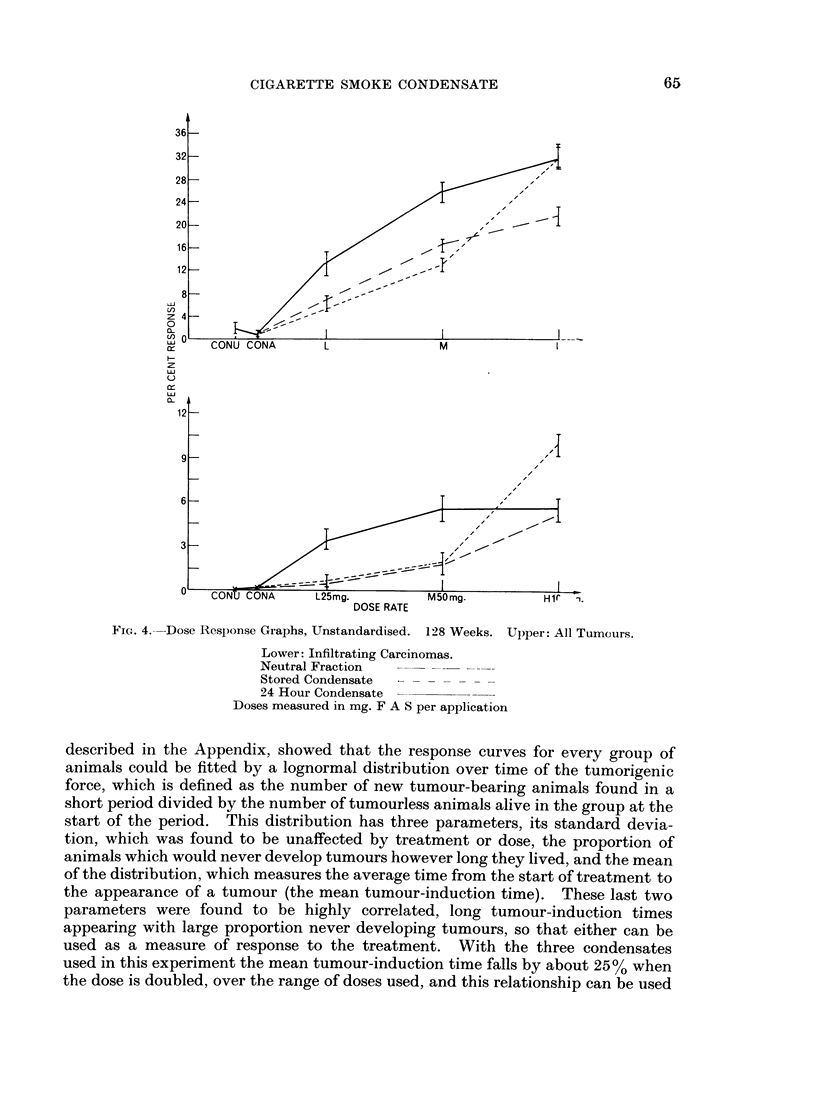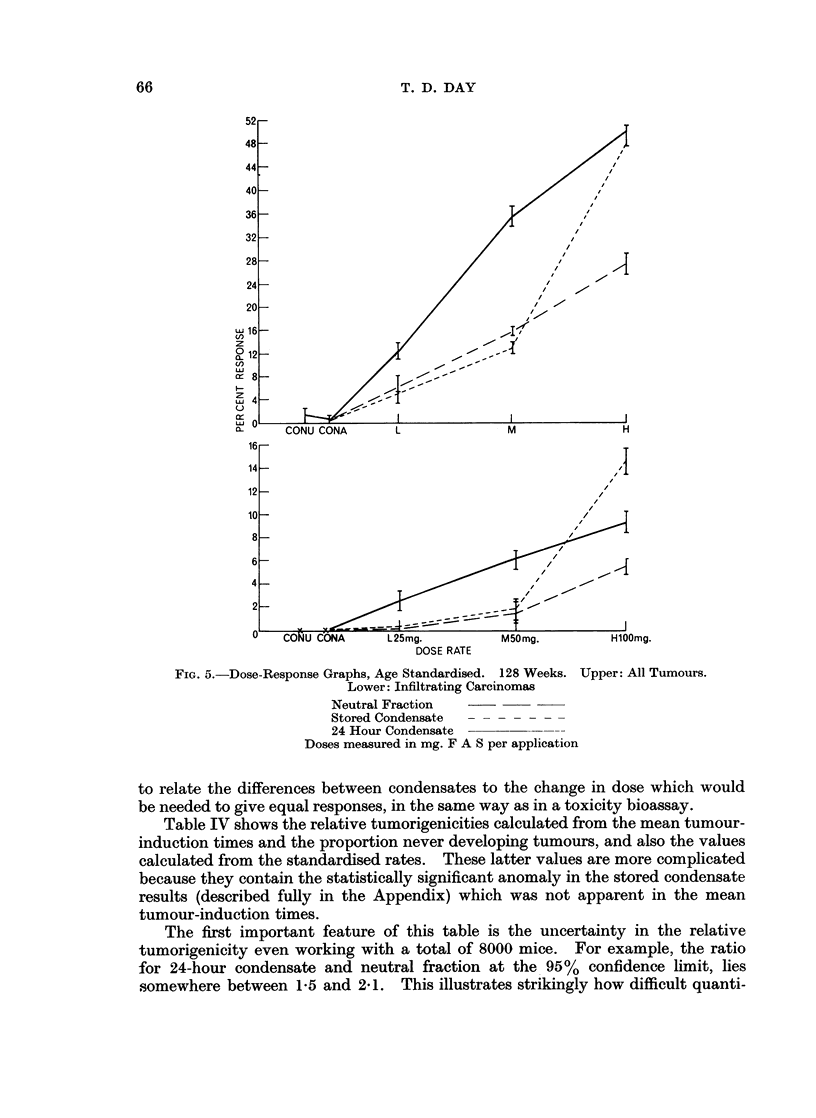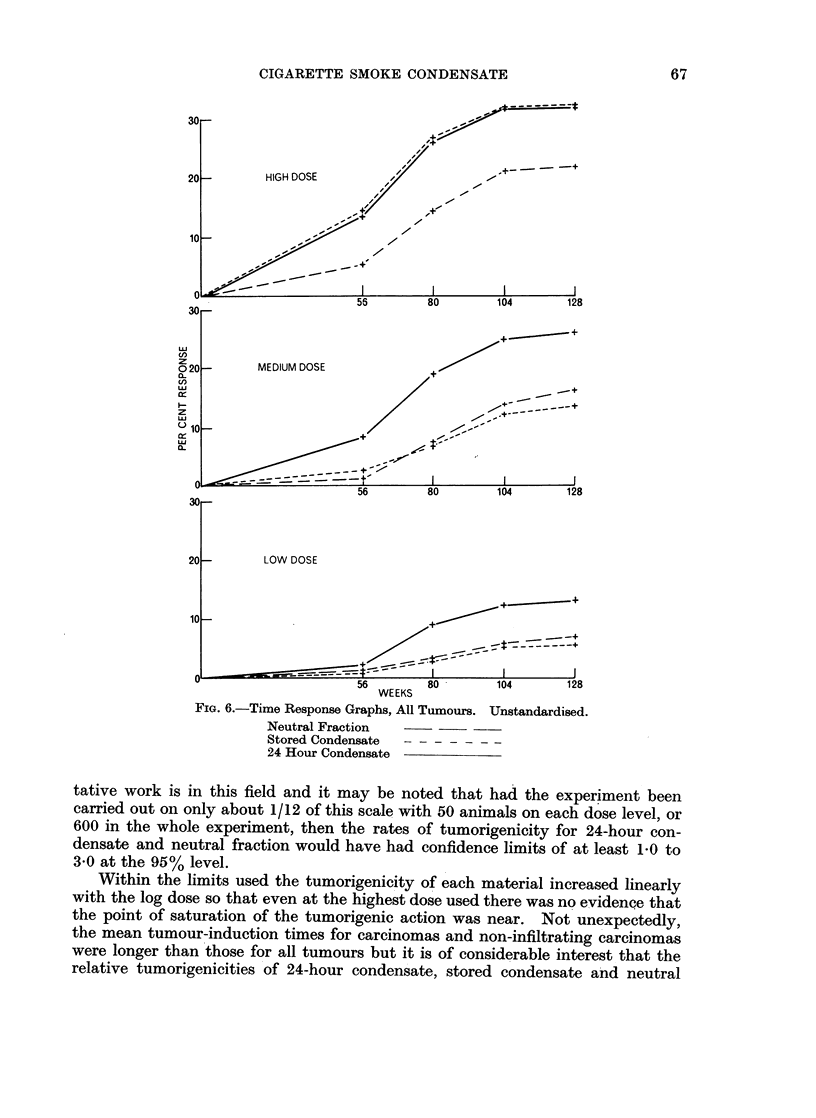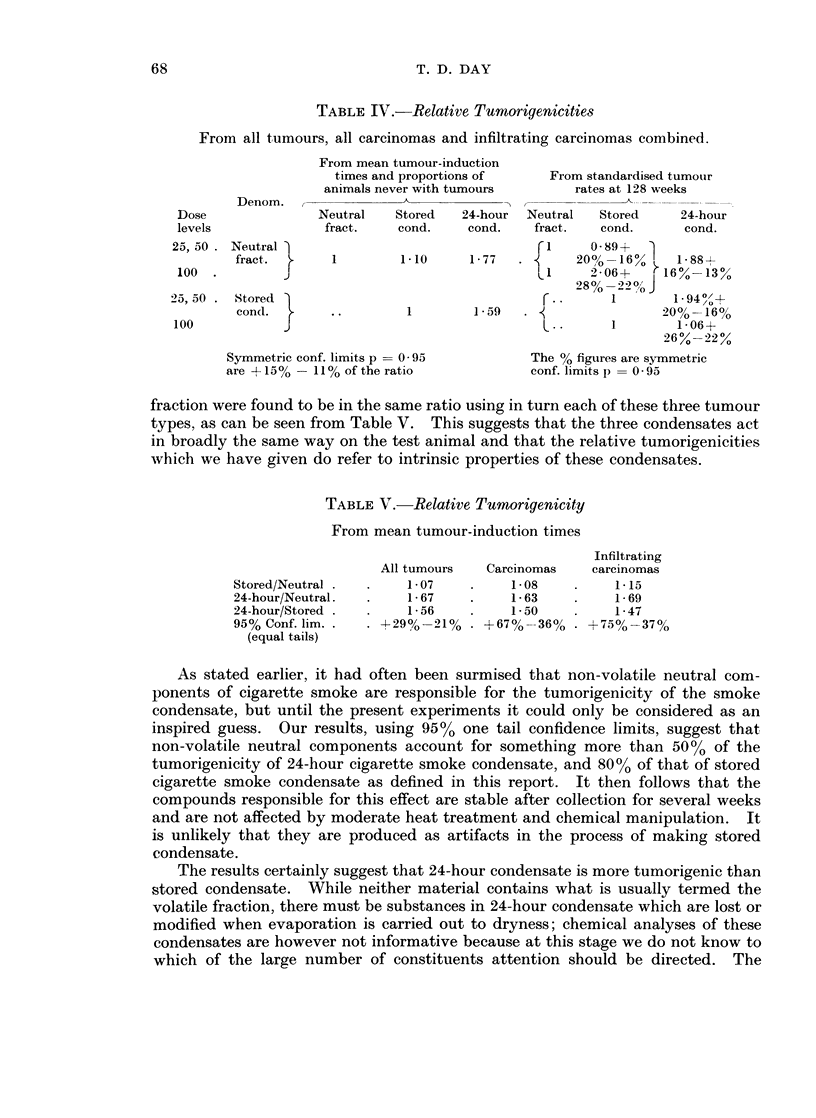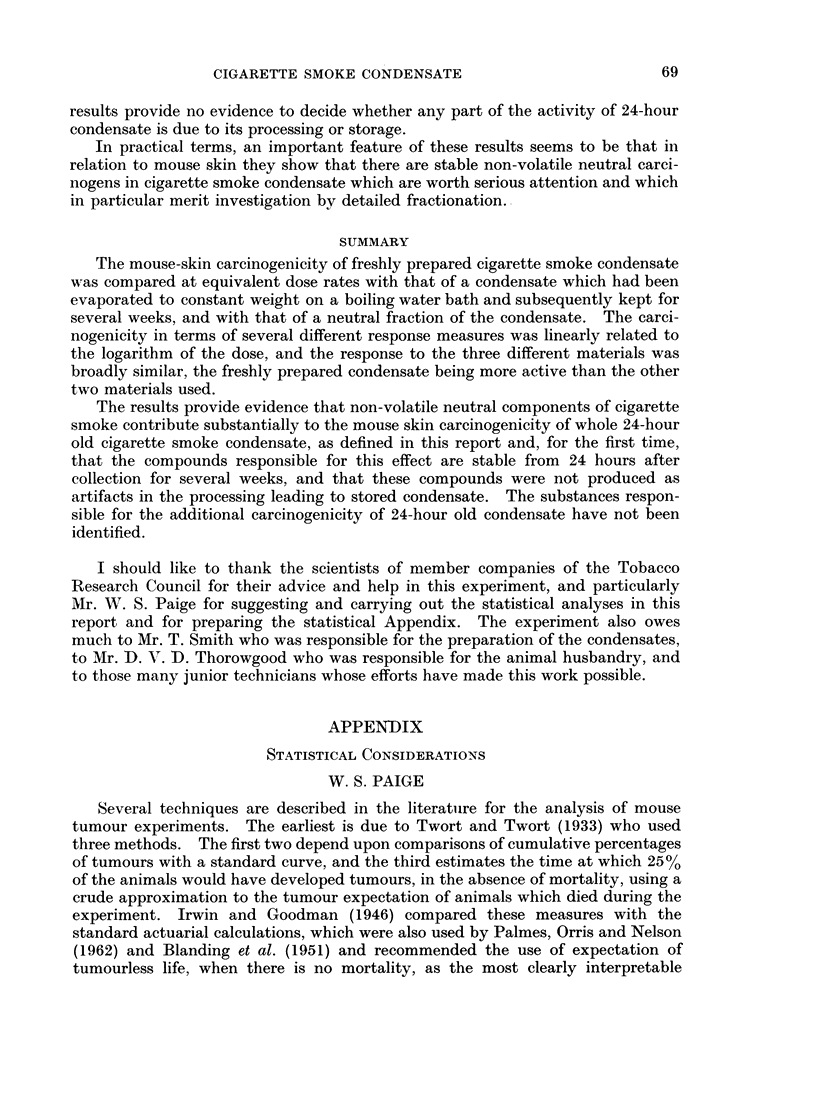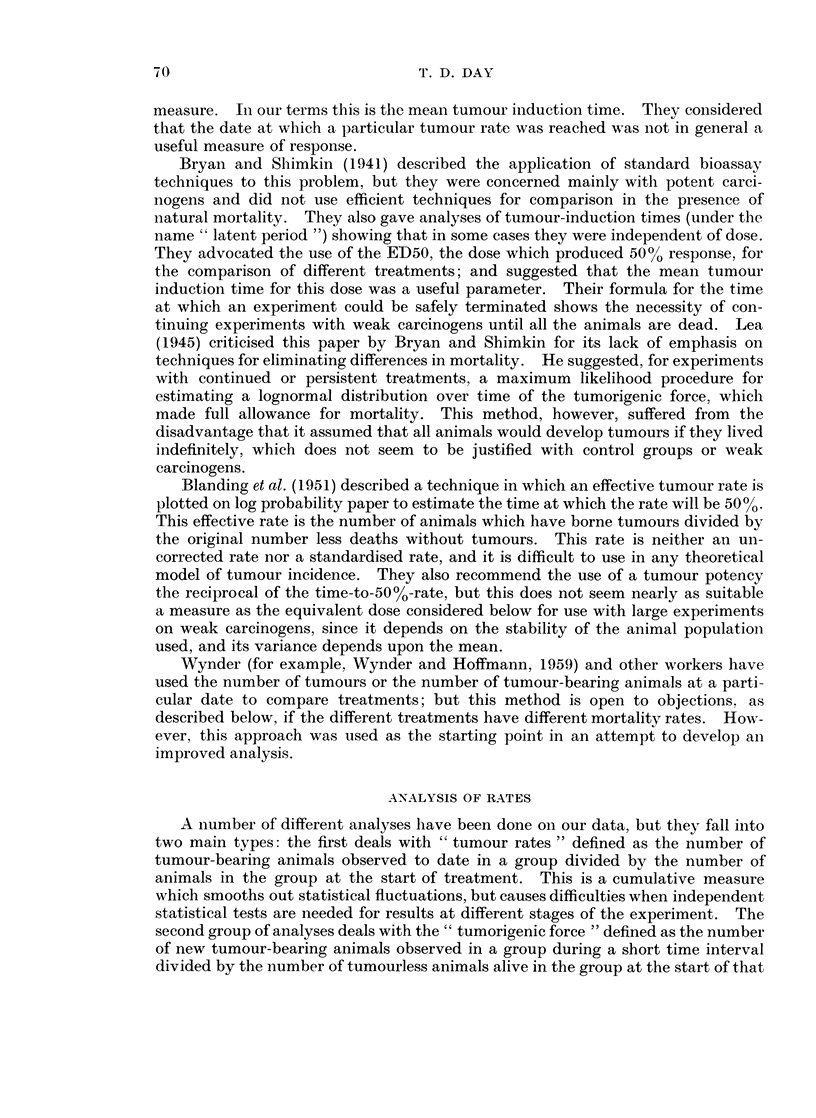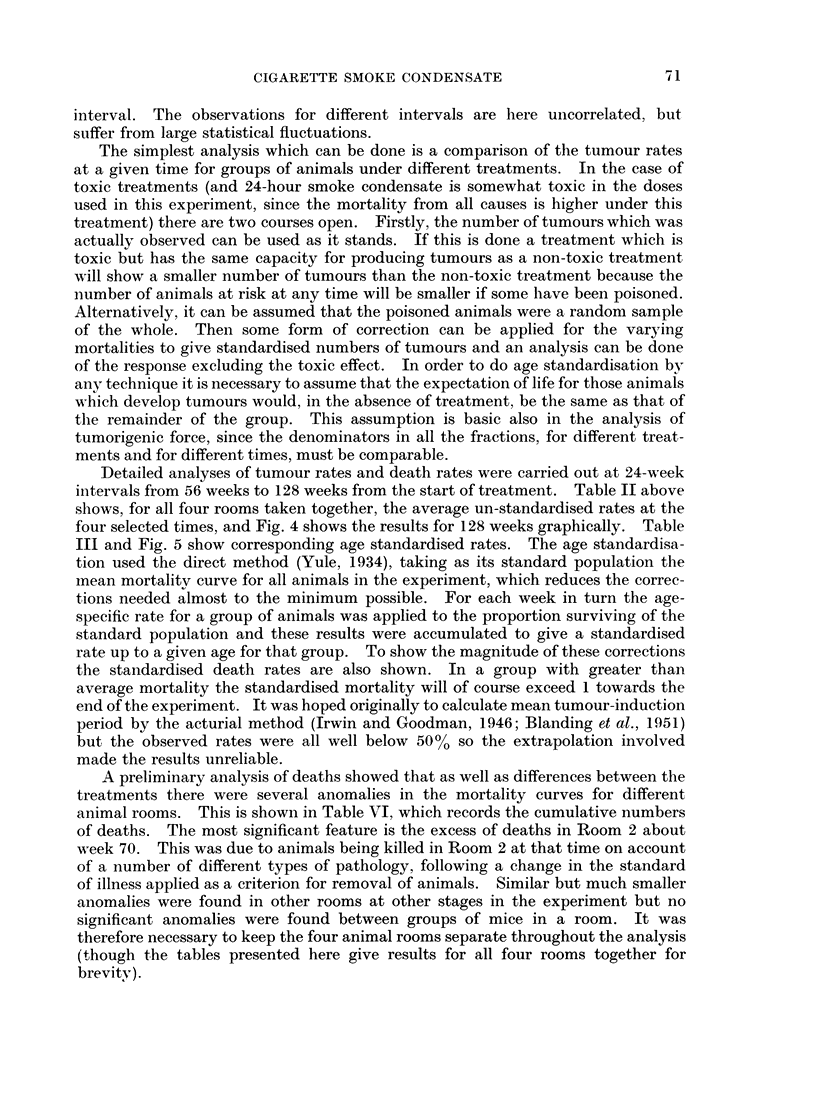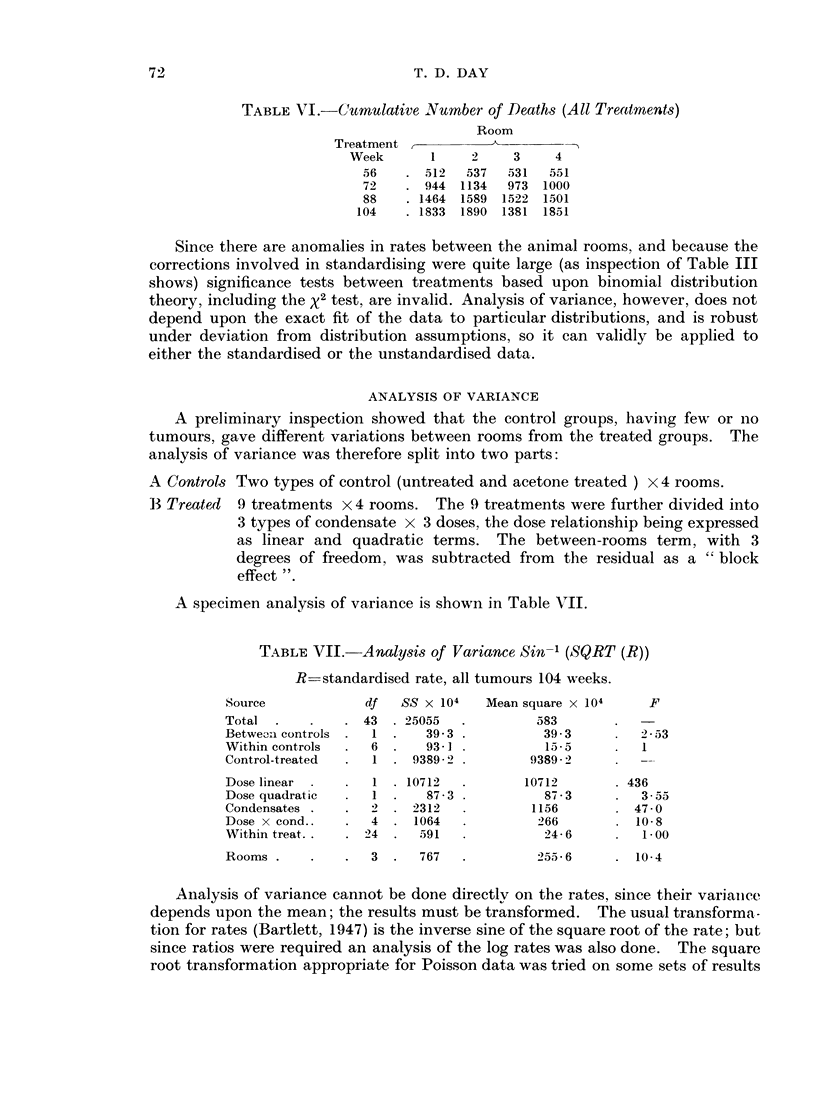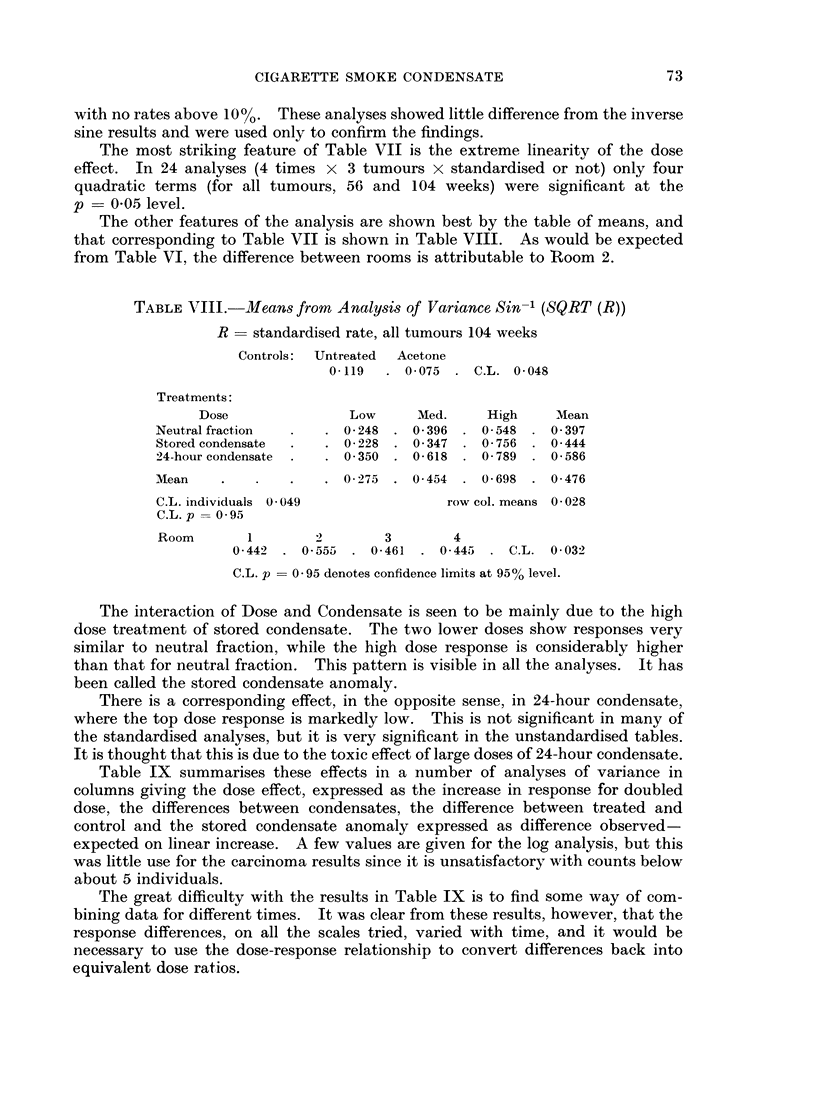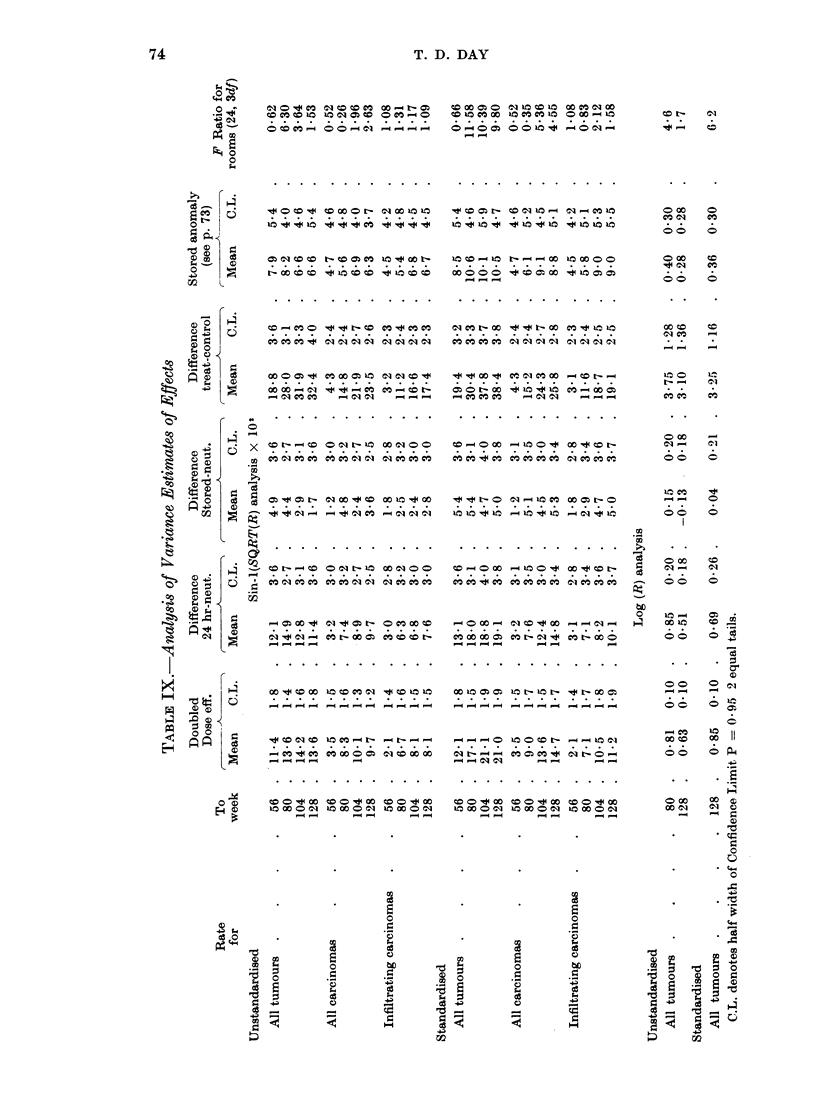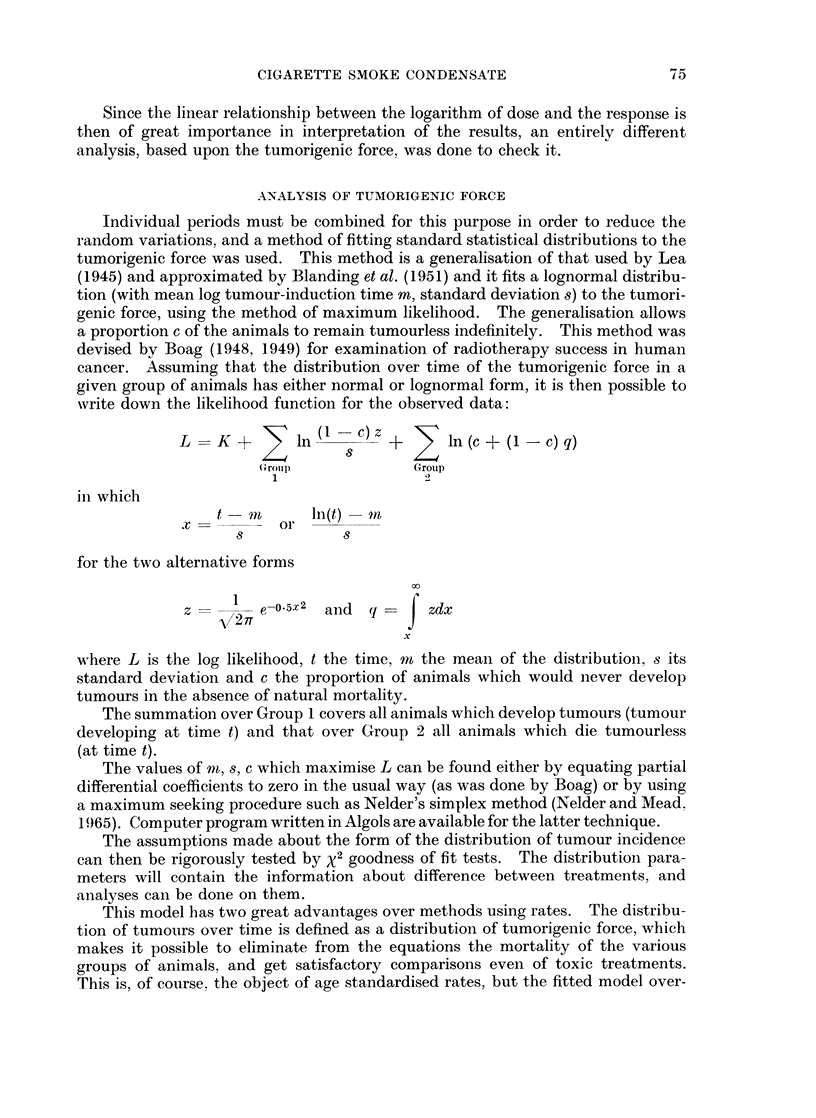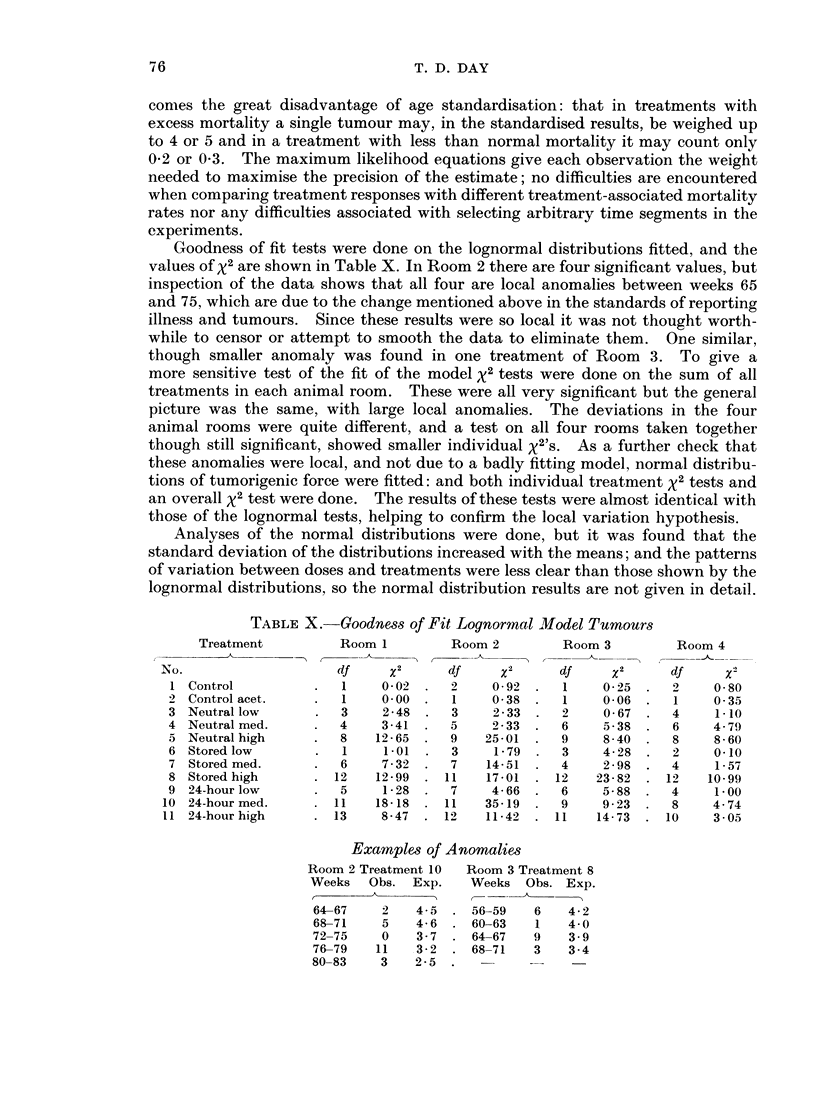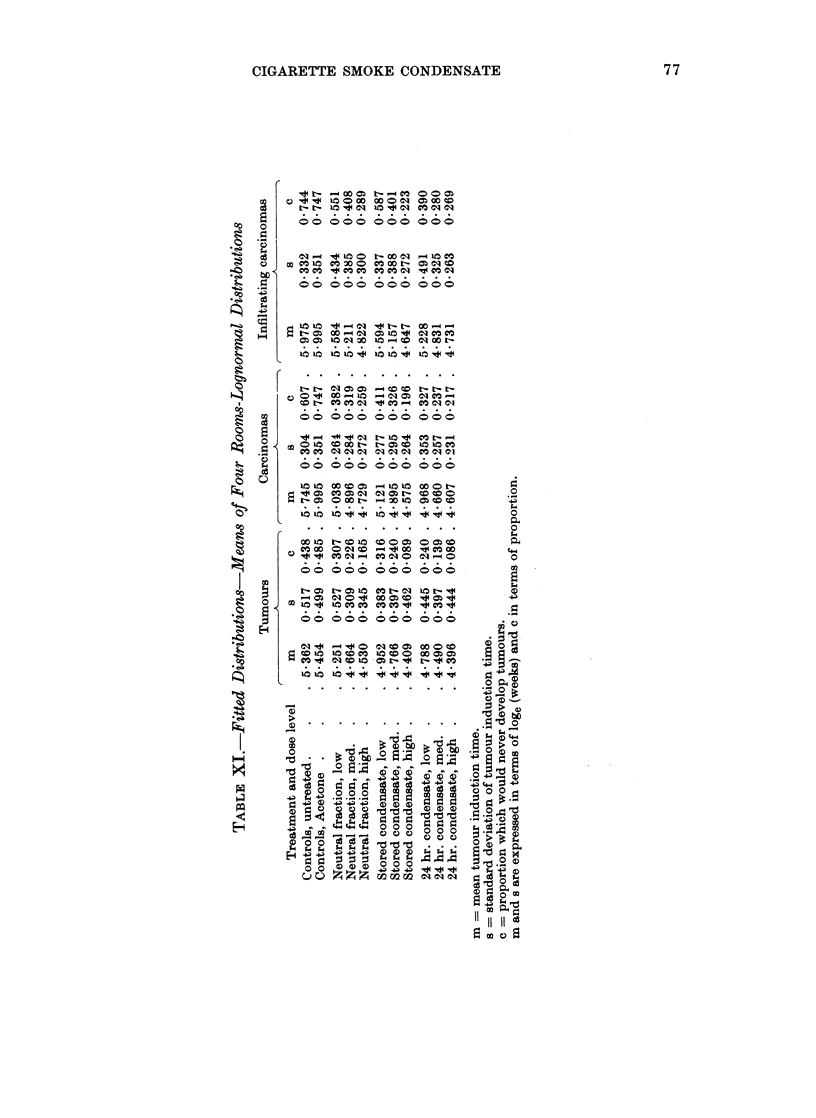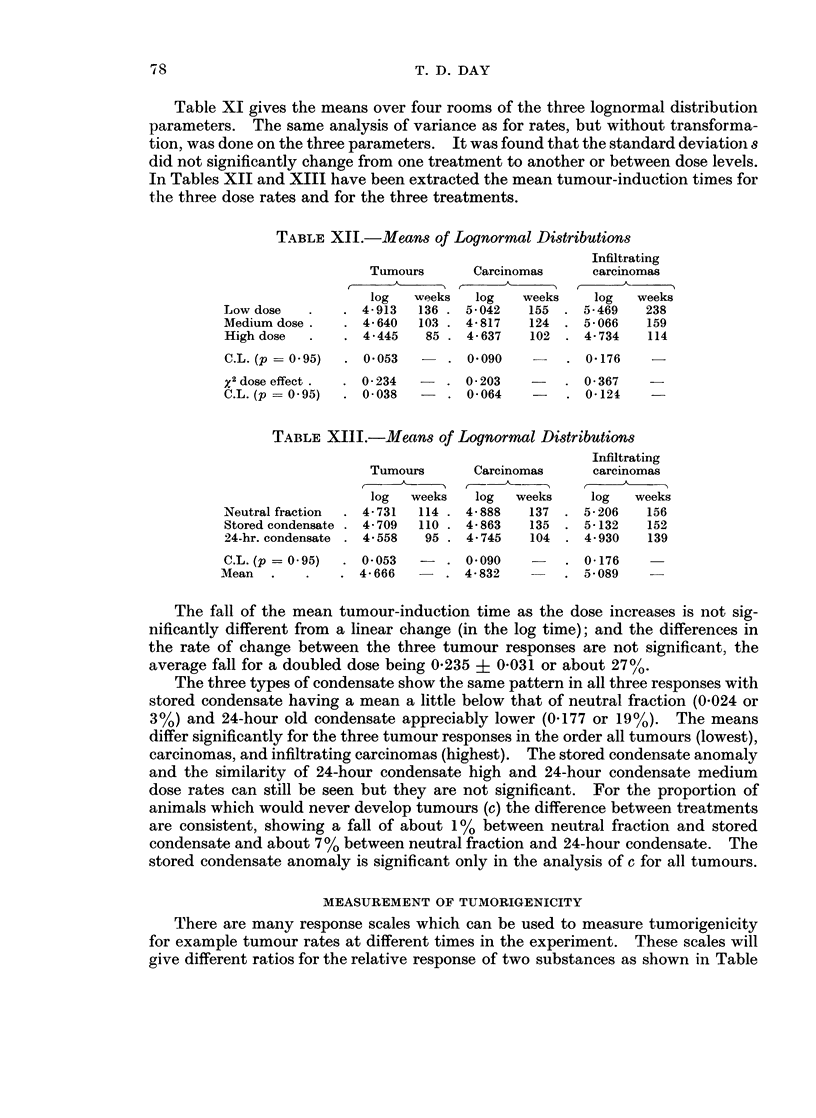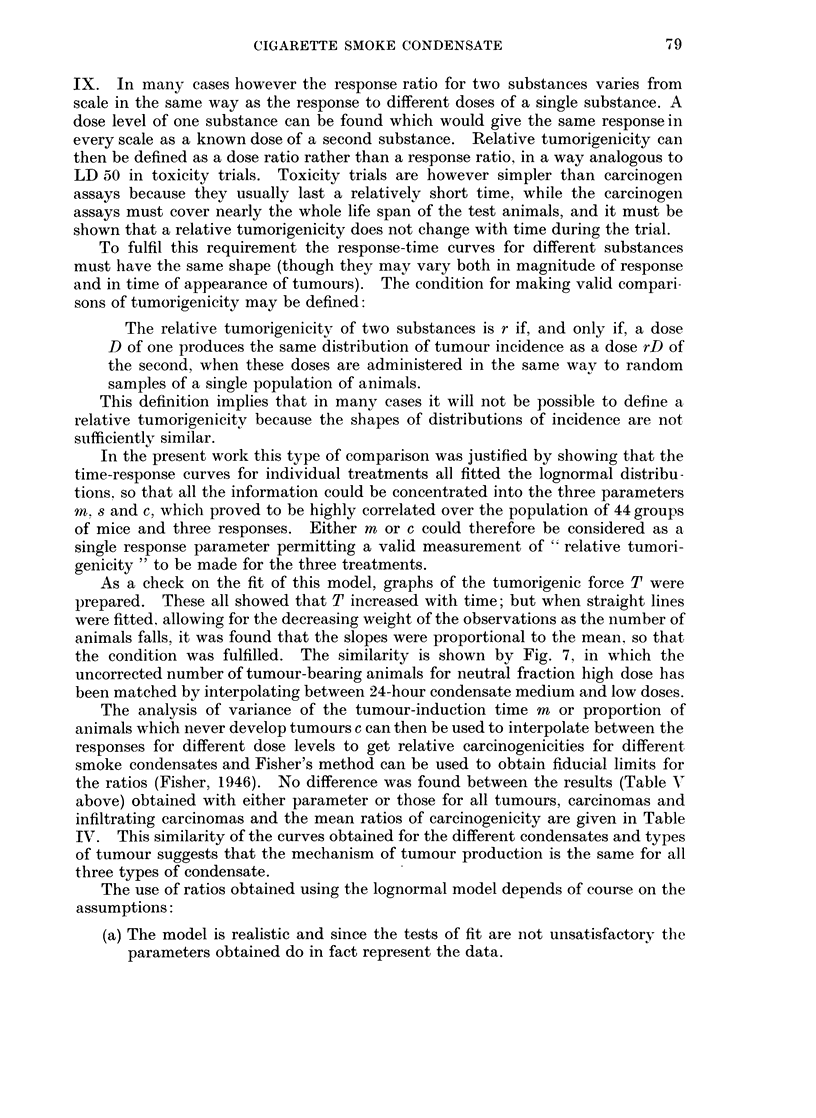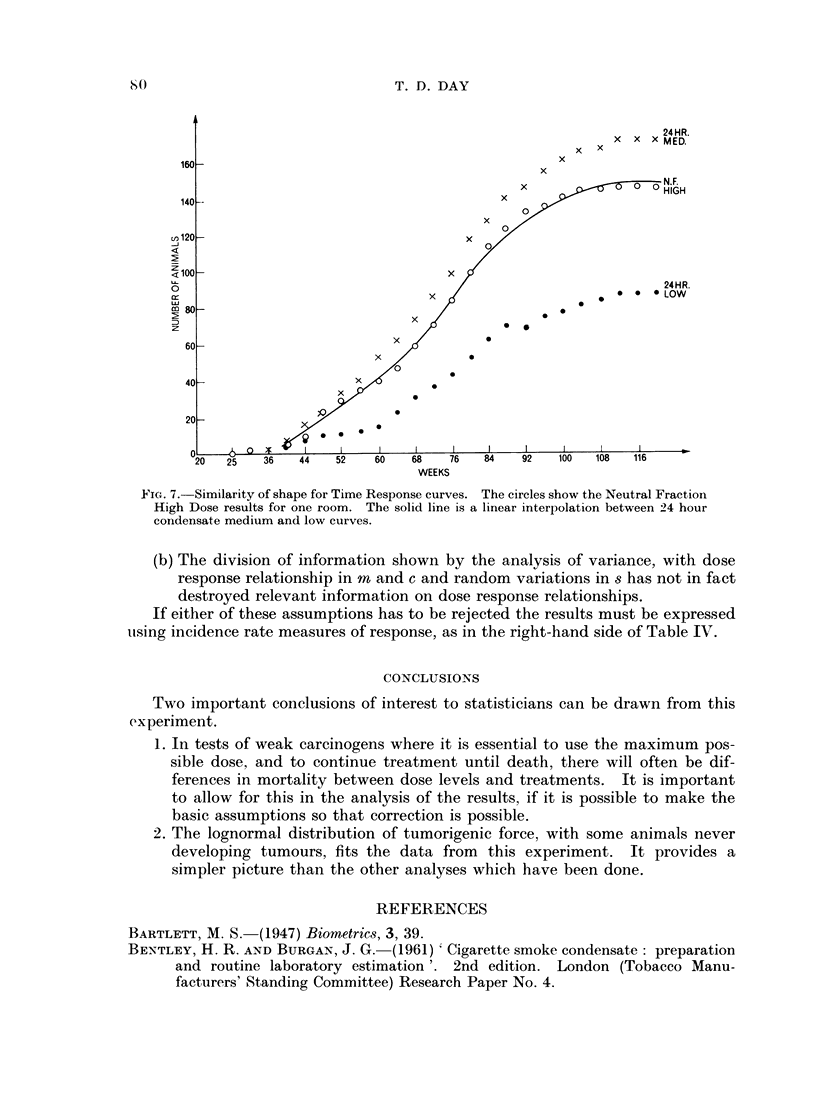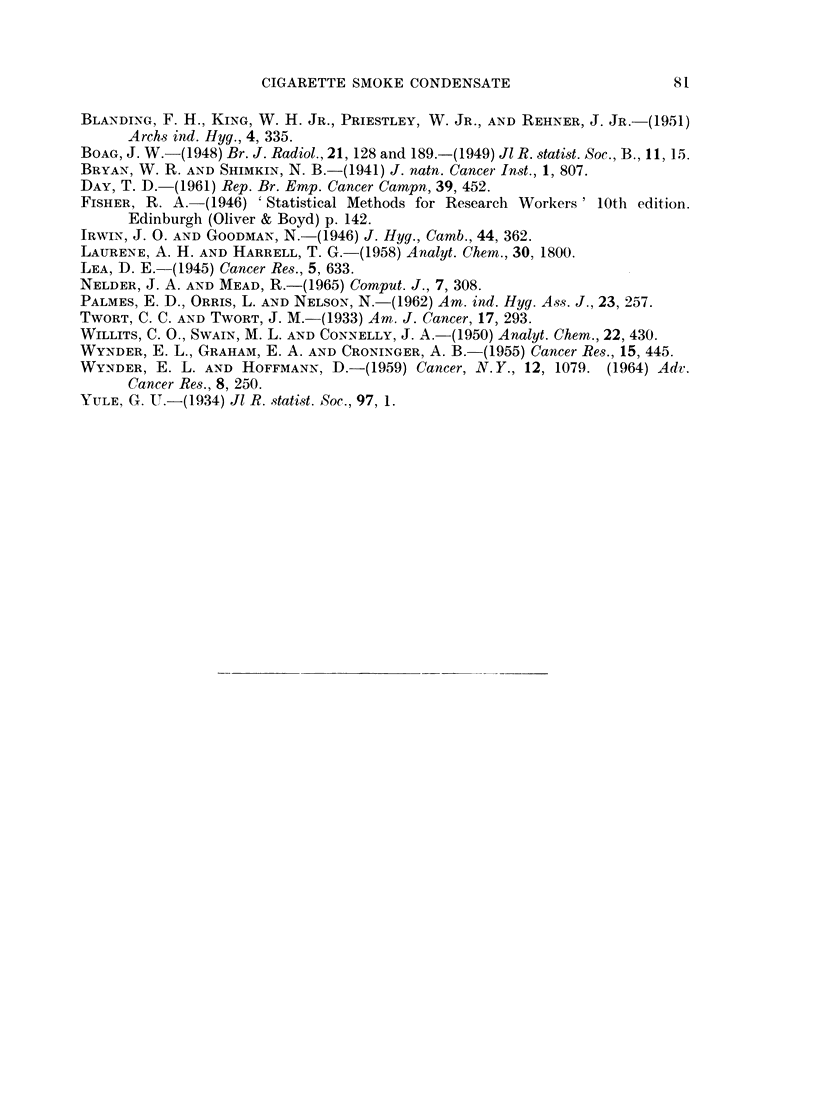# Carcinogenic action of cigarette smoke condensate on mouse skin.

**DOI:** 10.1038/bjc.1967.8

**Published:** 1967-03

**Authors:** T. D. Day

## Abstract

**Images:**


### CARCINOGENIC ACTION OF CIGARETTE SMOKE CONDENSATE ON MOUSE SKIN

### AN ATTEMPT AT A QUANTITATIVE STUDY

T. D. DAY

*From the Tobacco Research Council Laboratories, Harrogate*

Received for publication September 28, 1966

IT has been known for some years that when cigarette smoke is condensed and the condensate subsequently dried and stored it forms a " tar "-like substance which when painted on the backs of certain strains of mice gives rise to epithelial tumours (literature reviewed by Wynder and Hoffmann, 1964). Among all the efforts which have been made in recent years to study the carcinogenic properties of cigarette smoke experimentally, this still remains the salient phenomenon; cigarette smoke condensate undoubtedly has the property of a complete carcinogen for epithelial tissue of laboratory animals. The fact that dried cigarette smoke condensate possesses this property has led to the belief that the agents in cigarette smoke condensate principally responsible for its mouse skin carcinogenicity are likely to be stable non-volatile compounds. This belief in turn has formed the basis for the suggestion by some workers (notably Wynder and Hoffmann (1964)) that reduction of total cigarette smoke condensate and reduction of agents in the condensate responsible for mouse skin carcinogenicity might be useful steps to take.

However, up to the end of 1962 when work was started at these laboratories, little account had been taken of the possible contribution to mouse skin tumorigenicity of even semi-volatile components of cigarette smoke condensate. Further, the possibility had not yet been examined that the processes, particularly of drying, involved in preparing smoke condensate for skin application, might have either decreased its tumorigenicity through the destruction of unstable compounds, or alternatively increased this tumorigenicity by the production of carcinogens which were not present in freshly prepared condensate. The effect of storing " tar " for 6 months instead of 1 month had been studied (Wynder, Graham and Croninger 1955), but no work had previously been reported on " tar " less than 1 month old. Neither had an attempt been made by large scale bioassay techniques to identify the important classes of tumorigenic compounds in cigarette smoke. In other words it was not known with reasonable certainty, when the work described in this report was started, whether stable non-volatile constituents of cigarette smoke condensate were important or trivial contributors to its mouse skin tumorigenicity. The general aim of the work to be described was to investigate this question on a scale sufficient to give reliable answers and to investigate further the suggestion (Wynder and Hoffmann, 1959; Day, 1961) that polycyclic aromatic hydrocarbons, or some other neutral constituents, might account for much of the tumorigenic activity of cigarette smoke condensate.

It is obvious that the mouse skin painting test is unsuitable for detecting the effects of constituents with very low boiling points. These are difficult to isolate and keep in solution in the usual organic solvents at room temperature; it is impossible to treat mice with very cold materials and in any case the natural body heat of the animals would lead to rapid loss of material with a very low boiling point, probably before the skin could absorb it. However, it is possible to compare a condensate containing semi-volatile substances, such as are retained in a low boiling solvent, with one from which these substances have been evaporated. Accordingly it was decided to compare the mouse skin tumorigenicity of acetone solutions of:

(1) Whole cigarette smoke condensate, applied as quickly as possible after collection while using standard collection traps, hereafter referred to as " 24-hour condensate ".
(2) Whole cigarette smoke condensate which had been evaporated to constant weight and stored for at least one month before application, hereafter referred to as " stored condensate ".
(3) The neutral fraction from whole smoke condensate, after storage for at least one month.

with

(4) Untreated animals and animals treated with acetone only, kept as controls.

Experiments by previous workers in this field had used relatively small numbers of animals, so that statistical analysis of the results had shown significant differences only for large effects. It was further recognised that the yields of tumours from the condensate treatments might be low and that the differences between the treatments might be small. Accordingly, the present experiment was conducted on a scale considerably larger than had previously been attempted, and with a degree of attention to detail not before attained, in order to be able to make the necessary quantitative comparisons acceptably precise.

### MATERIALS AND METHODS

### *Experimental design*

A maximum response of about 30% of animals developing tumours was expected at the highest practicable dose. The experiment was therefore planned to enable a difference between 30% tumour rate and 20% tumour rate to be detected at the $p = 0{\cdot}02$ significance level in four tests out of five. To give some estimate of the dose-response relationship and to guard against the possibility of choosing the wrong dose, it was planned to divide those animals treated with condensates equally between three dose levels with dose ratios 1 : 2 : 4. A total of about 8000 mice was needed in order to achieve these aims.

Each of the four treatments was divided into three sections. Mice in each of the three sections of the condensate treatments received different doses; mice in one section of the control treatment were treated with acetone alone, mice in the remaining two sections were untreated. The experiment was housed in four animal rooms, using mice supplied at different times; each section was equally represented in each room and in every row of animal boxes in each room. The difference between animal rooms proved to be important, so for most purposes the experiment could be regarded as 40 groups of 165 mice each and four groups of 330 mice.

*Mice*

The mice were virgin females of a specific pathogen-free, albino strain supplied by Dr. W. G. Davey of the Pharmaceuticals Division, Imperial Chemical Industries Ltd. This strain, as the present paper shows, combined resistance to nicotine with sensitivity to skin carcinogens. Male mice were not used, being more pugnacious and so liable to skin damage. The mice, 4–6 weeks old on receipt, were transferred to sterile boxes and allowed to acclimatise for one month before skin applications were made. Four groups of 2100 animals, received at intervals of three months, were used. Of each group, a total of 1980 were used for treatment, 100 for calibration tests with standard carcinogens as described below, and the remainder for replacement of any animals found to be unsuitable: as for instance being unduly small or having been injured in transit. Mice were not replaced after skin treatment had commenced, and treatment was continued until the animals died or had to be removed due to illness.

Each group of 1980 mice was housed in a separate air-conditioned room, kept at 20–21° C., the aim being to separate the experiment into four equal units in order to limit the spread of any adventitious infective disease. In the event, no epidemic disease occurred and the death rates of the colonies in each of the four rooms although not identical were similar.

Mice were identified by ear punching and kept on sterile sawdust, three in a box, in sterilised galvanised iron boxes, being transferred to clean boxes twice a week. Drinking water in sterile bottles and heat sterilised (30 min. at 63° C.) Oxoid Breeding Diet pellets were provided *ad libitum.*

An area 1·5 cm. wide from nape of neck to base of tail of each mouse was kept clipped. Sesame oil (generally regarded as non-carcinogenic and non-promoting) was used as clipper lubricant.

*Dosing procedure*

A preliminary toxicity test of nicotine-containing condensate was undertaken in order to ascertain the highest practicable dose-level for long-term application. Dosing by the standard procedure described below was continued for six weeks; acute toxicity was estimated by observing whether the animals were ill immediately after dosing, and chronic toxicity by observing whether the animals gained weight. The results indicated that a top level equivalent to 100 mg. non-volatile whole smoke condensate (as defined below) per application was suitable, after habituation, for long-term treatment with acceptably low mortality. Acetone solutions of test materials, prepared as described later, were therefore applied at dose levels equivalent to 100, 50 and 25 mg. of non-volatile whole smoke condensate per application, the highest dose level being commenced only after habituation for one month at 75 mg. dose per treatment.

Skin applications were made three times a week, with a foot-controlled automatic pipette, delivering for all treatments a standard volume of 0·3 ml. through silicone rubber tubing; pipettes were checked and re-calibrated if necessary daily. The standard volume of fluid was applied evenly on the clipped area.

The reaction to skin carcinogenesis of a sample from every batch of mice received at three-monthly intervals was checked by treatment with acetone solutions (0·1% w/v) of 1,2,5,6-dibenzanthracene and 3,4-benz(a)pyrene, 0·019 ml. of solution being applied three times per week. Applications to one-half of each sample of mice was stopped after 10 weeks, treatment of the remainder continuing for 25 weeks. All these animals developed tumours in a few months, no great differences being observed between the batches.

*Pathology at death and diagnosis of tumours*

A full post mortem examination was performed on each mouse, whether bearing skin tumours or not, and a suitable sized piece of tissue was taken from any organ showing abnormality. Slides and post mortem descriptions were interpreted by the same pathologist throughout. All tumours were examined histologically and their nature recorded, a clear distinction being made between tumours occurring in the treated area of the skin and elsewhere. A total of 45 animals were recorded as dying from nicotine poisoning; their records were eliminated from all results. A summary of the pathology recorded at death for the remaining mice is given in Table I.

TABLE I.—*Pathology Recorded at Death*

All 7875 mice in the experiment (4 × 1980 less 45 deaths due to poisoning)

| | Predominant pathology at time of death | Per cent of all mice with this pathology |
|---|---|---|
| 1. | Hepato nephritis . . . . | 23·0 |
| 2. | Malignant lymphoma . . . . | 22·6 |
| 3. | Inflammation of uterus and other miscellaneous inflammation | 12·2 |
| 4. | Dermatitis and skin sepsis . . . | 8·7 |
| 5. | Malignant tumours (not on painted area and including sarcomas) | 6·7 |
| 6. | All haemorrhages . . . . | 6·7 |
| 7. | All benign tumours (haemangiomas, papilloma of painted area and adenomas other than of lung) | 5·0 |
| 8. | Due to experiment (cause not determined, etc. but *not* nicotine poisoning) | 4·4 |
| 9. | Adenoma of lung . . . . | 3·6 |
| 10. | Miscellaneous (general) . . . | 3·2 |
| 11. | Malignant tumours on painted area . | 3·0 |
| 12. | Staphylococcal osteomyelitis . . | 0·9 |

Apart from skin sepsis, which was more common in the high dose groups, the pathology was not particularly related to treatment and the incidence of tumours, other than those of the painted area, did not vary significantly with dose or treatment. Any animal considered at any time by the animal superintendent to be irrecoverably ill was killed, as were mice which escaped from their boxes.

For cancers of the skin in the treated area, particular attention was paid to making the diagnosis of malignancy independent of subjective factors. The strict criterion of malignancy adopted was penetration by the epidermal tumour between the muscle fibres of the panniculus carnosus (" muscle-infiltrating carcinoma ") as shown in Fig. 1. Tumours which had the subjective appearance of infiltrating cancer but which did not fulfil this strict criterion of muscle penetration were separately classified (" carcinoma not infiltrating muscle "). The rare occurrence of a subcutaneous sarcoma or haemangiendothelioma in the treated area was also noted.

The date of first appearance of a papilloma was noted on a record card, kept for each animal throughout the experiment. Tumour-bearing animals were normally killed when the tumour appeared to have become malignant, as judged by the pathologist pinching up the skin and assessing the degree of infiltration on its under surface. The date of death was then recorded as the date of appearance of a carcinoma, if one was found by histology.

*Cigarettes*

Plain cigarettes (length 70 mm., circumference 25·3 mm., average weight 1·125 g.) were specially manufactured from a composite blend of flue-cured tobacco representing the major plain cigarette brands smoked in the United Kingdom, packed in batches of 50 in vacuum-sealed tins and stored at 4° C. before use.

*Smoking procedure*

The automatic smoking machine used operates by connecting each of 24 cigarettes, secured in holders situated round a revolving disc, in turn to a source of vacuum, the unlighted end of each cigarette being open to atmosphere between puffs (Fig. 2). Cigarettes were lighted by an electrically heated coil. When individual cigarettes had reached an estimated butt length of 20 mm., the butts were removed and replaced with fresh cigarettes.

Automatic smoking constants were chosen to simulate the manner used by the average cigarette smoker in the United Kingdom (Bentley and Burgan, 1961):

Puff volume, 25 ml.; Puff duration, 2 seconds;
Puff interval, 1 minute; Butt length, 20 mm.

*Whole smoke condensate (WSC)*

Cigarette smoke was collected in four glass traps connected in series (Fig. 3), cooled in acetone/crushed solid carbon dioxide. Traps 3 and 4 each contained glass helices (100 g., 4 mm. diam. single turn). On completion of smoking, traps were allowed to attain room temperature, condensed smoke was washed from the traps and connecting tubes with acetône (about 900 ml.), the washings filtered through glass wool and an aliquot of the combined filtrate taken to check non-volatile whole smoke yield by determination of nicotine. The average yield of nicotine was 1·61 mg./cigarette, range 1·30–1·91 mg./cigarette.

EXPLANATION OF PLATES

FIG. 1a.—Malignant infiltration of the subcutaneous tissue by epidermal cancer cells, but these have not yet penetrated the muscle. H. and E. × 80.

FIG. 1b.—Epidermal cancer cells which have infiltrated between the fibres of the panniculus carnosus muscle. This is the usual criterion for the diagnosis of malignancy. H and E. × 190.

FIG. 2.—Smoking machine.

1a

1b

Day.

3

2

Day.

FIG. 3a.—Smoke condensation system. The arrow on the right shows the exit to the smoking machine, via a cotton wool filter. The two right hand traps contain Fenske helices (single turn 4 mm. diam.) and all four Dewar flasks are filled with solid carbon dioxide and acetone.

*Non-volatile whole smoke condensate (NVWSC)*

Solvent was removed from the acetone solution of WSC in a weighed flask, using a rotary evaporator on a boiling water bath with a water suction pump operating at a vacuum of 10 inches of mercury; evaporation was continued until the non-volatile residue attained constant weight. The average yield was 21·5 mg./cigarette, range of 17·7–24·8 mg./cigarette. All doses of all materials applied to animals were expressed in terms of the weight of NVWSC determined in this way, each individual dose, irrespective of weight, being delivered in the standard volume (0·3 ml.) of solution. The average yield of NVWSC over a four-week period was used to compute the number of cigarettes to be smoked for both 24-hour condensate solutions and for the stored condensate and neutral fraction required during the subsequent four-week period.

*Stored condensate*

NVWSC collected over four weeks was combined, stored at —29° C. for a further four weeks, dissolved with constant stirring in acetone/water (9 : 1 v/v) and the solution diluted to the appropriate volume with the same solvent.

*24-hour condensate*

The acetone solution of WSC from the calculated number of cigarettes was sampled for nicotine content to check the actual WSC yield and concentrated in a rotary evaporator (water bath temperature 40° C. and the full vacuum of a water suction pump), until it reached the concentration required for application at the highest dose rate. Aliquots of the concentrate were then taken for dilution with acetone/water (9 : 1 v/v) to provide solutions for the two lower dose levels.

Fig. 3b.—Details of Trap.

*Neutral fraction*

Redistilled peroxide-free Reagent Grade diethyl ether was treated with sodium to remove fluorescent material. The smoke condensate from a known number of cigarettes (about 2000) was washed from the traps into a separating funnel with ether (1 l.) and hydrochloric acid (" Analar " 2 N, 900 ml.), the mixture was shaken, allowed to separate and the aqueous layer drawn off into a second separating funnel. After further extractions with hydrochloric acid (2 × 200 ml.,

5 × 100 ml.), the combined acid extracts were washed with ether (2 × 100 ml.), and the ether washings returned to the ether solution left in the first separating funnel. The combined ether solution was then vigorously shaken with potassium hydroxide solution (3% w/v, 200 ml.), allowed to stand for 1½ hours, the aqueous layer drawn off and further extractions carried out with potassium hydroxide solution (1 × 200 ml., 5 × 100 ml.); little emulsification occurred after the first extraction and the two layers separated without difficulty. The final ether solution was dried (anhydrous magnesium sulphate), filtered and the solvent removed in a rotary evaporator (water bath at 40° C. and a water suction pump)

The average yield of neutral fraction was 6·68 mg./cigarette, range of 5·00–7·88 mg./cigarette; the condensate yield of each batch was checked by estimating the nicotine content of a suitable aliquot of the combined acid extracts. Batches of neutral fraction produced in a four-week period were combined and the corresponding NVWSC equivalent calculated from the total number of cigarettes smoked and the average yield of NVWSC obtained for stored smoke condensate over that period of four weeks. For dosing, the combined neutral fraction was dissolved in acetone/water (9 : 1 v/v).

*Nicotine assay*

Nicotine content was measured on aliquots of the condensate obtained from every individual smoking, to check on smoking performance and extraction efficiency, by the method of Willits, Swain and Connelly (1950), as modified by Laurene and Harrell (1958).

### DISCUSSION OF RESULTS

Table II shows for all animals the percentage rates (100 × No. affected/Initial No.) for death and incidence of three classes of tumours at four times during the experiment. No allowance has been made in these results for differences in mortality between treatments, and it can be seen that there are quite large variations in mortality from one treatment to another. To give a fair comparison of the treatments, independent of their toxicity, some form of age standardisation must be done on the assumption (which seems unavoidable in experiments of this type) that animals which develop tumours would not, in the absence of treatment, have lived longer or died sooner than the others. The age standardised results, calculated in the way described in the Appendix are shown in Table III.

In order to attain the objectives outlined earlier, it was necessary to discover if the data would permit a valid comparison to be made of the tumorigenicities of the three materials under test. It was not the intention in planning the experiment to investigate the mechanisms of carcinogenesis, for example the relative contributions in each material from tumour initiators and tumour promoters, but unless the three materials act in broadly similar ways there can be no valid basis for comparing their relative tumorigenicities. Graphical representation of the data, exemplified by Fig. 4, 5 and 6, in which no account is taken of the statistical confidence limits of the points, suggested an anomaly in that at the low and medium dose levels stored condensate and neutral fraction appeared to have similar activities, each different from 24-hour condensate, whereas at the high dose level stored condensate and 24-hour condensate appeared to have similar activities, each different from neutral fraction. The statistical analysis, which is

TABLE II—*Unstandardised Percentage Response*

| Treatment and Dose level | Deaths Weeks | | | | Tumour bearing animals Weeks | | | | Carcinoma bearing animals Weeks | | | | Animals bearing infiltrating carcinomas Weeks | | | |
|---|---|---|---|---|---|---|---|---|---|---|---|---|---|---|---|---|
| | 56 | 80 | 104 | 128 | 56 | 80 | 104 | 128 | 56 | 80 | 104 | 128 | 56 | 80 | 104 | 128 |
| Controls, Untreated | 25·70 | 61·03 | 92·72 | 99·55 | 0·23 | 0·99 | 1·59 | 1·82 | 0·00 | 0·00 | 0·08 | 0·03 | 0·00 | 0·00 | 0·00 | 0·00 |
| Controls, Acetone | 25·04 | 61·76 | 92·41 | 99·39 | 0·00 | 0·30 | 0·76 | 0·91 | 0·00 | 0·00 | 0·00 | 0·00 | 0·00 | 0·00 | 0·00 | 0·00 |
| Neutral fraction, low | 28·07 | 64·34 | 92·87 | 99·70 | 1·37 | 3·34 | 5·92 | 6·98 | 0·00 | 0·30 | 1·06 | 1·21 | 0·00 | 0·30 | 0·46 | 0·46 |
| Neutral fraction, med. | 24·39 | 61·06 | 92·88 | 99·70 | 1·06 | 7·58 | 13·64 | 16·06 | 0·30 | 1·06 | 3·79 | 4·09 | 0·15 | 0·45 | 1·82 | 1·82 |
| Neutral fraction, high | 28·64 | 68·94 | 95·45 | 100·00 | 5·30 | 14·85 | 21·36 | 21·97 | 0·45 | 3·79 | 7·12 | 7·73 | 0·30 | 2·73 | 4·70 | 5·15 |
| Stored condensate, low | 24·89 | 58·73 | 92·41 | 99·85 | 0·61 | 3·19 | 5·31 | 5·77 | 0·00 | 0·30 | 0·76 | 1·06 | 0·00 | 0·15 | 0·46 | 0·61 |
| Stored condensate, med. | 26·36 | 63·64 | 92·12 | 100·00 | 2·42 | 6·82 | 12·27 | 13·33 | 0·00 | 1·67 | 3·33 | 4·39 | 0·00 | 0·76 | 1·52 | 1·82 |
| Stored condensate, high | 30·30 | 73·64 | 97·58 | 100·00 | 14·55 | 27·12 | 32·27 | 32·58 | 1·52 | 8·48 | 13·94 | 14·85 | 1·06 | 5·30 | 9·24 | 9·70 |
| 24 hr. condensate, low | 23·33 | 57·73 | 91·97 | 99·70 | 2·27 | 9·39 | 12·58 | 13·33 | 0·30 | 1·67 | 3·94 | 4·85 | 0·30 | 1·06 | 2·73 | 3·33 |
| 24 hr. condensate, med. | 30·30 | 70·15 | 96·67 | 100·00 | 8·03 | 19·09 | 25·00 | 25·91 | 0·61 | 4·39 | 9·39 | 10·30 | 0·45 | 2·88 | 4·85 | 5·30 |
| 24 hr. condensate, high | 32·36 | 71·20 | 99·19 | 100·00 | 13·75 | 26·54 | 31·88 | 31·88 | 0·81 | 4·53 | 10·36 | 11·17 | 0·32 | 2·91 | 5·18 | 5·50 |

TABLE III.—*Standardised Percentage Response*

| Treatment and Dose level | Deaths Weeks | | | | Tumour bearing animals Weeks | | | | Carcinoma bearing animals Weeks | | | | Animals bearing infiltrating carcinomas Weeks | | | |
|---|---|---|---|---|---|---|---|---|---|---|---|---|---|---|---|---|
| | 56 | 80 | 104 | 128 | 56 | 80 | 104 | 128 | 56 | 80 | 104 | 128 | 56 | 80 | 104 | 128 |
| Controls, untreated | 25·47 | 58·00 | 86·05 | 90·37 | 0·23 | 0·91 | 1·44 | 1·67 | 0·00 | 0·00 | 0·08 | 0·08 | 0·00 | 0·00 | 0·00 | 0·00 |
| Controls, Acetone | 24·73 | 59·64 | 87·10 | 90·44 | 0·00 | 0·30 | 0·76 | 0·76 | 0·00 | 0·00 | 0·00 | 0·00 | 0·00 | 0·00 | 0·00 | 0·00 |
| Neutral fraction, low | 28·68 | 63·58 | 90·90 | 94·54 | 1·37 | 3·34 | 6·22 | 6·68 | 0·00 | 0·30 | 1·06 | 1·06 | 0·00 | 0·30 | 0·46 | 0·46 |
| Neutral fraction, med. | 23·79 | 57·73 | 86·82 | 92·42 | 1·06 | 7·58 | 15·15 | 15·76 | 0·30 | 1·06 | 3·64 | 3·79 | 0·00 | 0·45 | 1·67 | 1·67 |
| Neutral fraction, high | 28·33 | 70·61 | 102·88 | 108·64 | 5·45 | 17·12 | 27·27 | 27·73 | 0·45 | 3·94 | 8·18 | 8·64 | 0·00 | 2·73 | 5·45 | 5·76 |
| Stored condensate, low | 24·28 | 55·54 | 84·07 | 88·92 | 0·61 | 2·88 | 5·31 | 5·61 | 0·00 | 0·30 | 0·76 | 0·76 | 0·00 | 0·15 | 0·46 | 0·46 |
| Stored condensate, med. | 25·91 | 64·85 | 92·42 | 98·94 | 2·42 | 6·67 | 11·97 | 13·03 | 0·00 | 1·67 | 3·18 | 3·79 | 0·00 | 0·76 | 1·67 | 1·82 |
| Stored condensate, high | 30·45 | 79·24 | 121·97 | 128·48 | 15·91 | 33·79 | 47·12 | 48·64 | 1·52 | 9·55 | 20·30 | 21·97 | 0·00 | 5·91 | 14·09 | 14·85 |
| 24 hr. condensate, low | 22·58 | 52·88 | 82·58 | 86·97 | 2·27 | 8·94 | 11·97 | 12·58 | 0·30 | 1·36 | 3·48 | 3·79 | 0·00 | 1·06 | 2·42 | 2·73 |
| 24 hr. condensate, med. | 30·76 | 73·94 | 109·70 | 116·52 | 8·48 | 22·88 | 33·79 | 35·76 | 0·61 | 5·00 | 11·67 | 13·64 | 0·00 | 3·18 | 5·45 | 6·36 |
| 24 hr. condensate, high | 33·66 | 77·18 | 128·64 | 132·69 | 15·37 | 34·63 | 50·49 | 50·49 | 0·81 | 5·02 | 16·99 | 20·71 | 0·00 | 3·40 | 7·61 | 9·55 |

All treatments applied three times a week until death.

FIG. 4.—Dose Response Graphs, Unstandardised. 128 Weeks. Upper: All Tumours.
Lower: Infiltrating Carcinomas.
Neutral Fraction
Stored Condensate
24 Hour Condensate
Doses measured in mg. F A S per application described in the Appendix, showed that the response curves for every group of animals could be fitted by a lognormal distribution over time of the tumorigenic force, which is defined as the number of new tumour-bearing animals found in a short period divided by the number of tumourless animals alive in the group at the start of the period. This distribution has three parameters, its standard deviation, which was found to be unaffected by treatment or dose, the proportion of animals which would never develop tumours however long they lived, and the mean of the distribution, which measures the average time from the start of treatment to the appearance of a tumour (the mean tumour-induction time). These last two parameters were found to be highly correlated, long tumour-induction times appearing with large proportion never developing tumours, so that either can be used as a measure of response to the treatment. With the three condensates used in this experiment the mean tumour-induction time falls by about 25% when the dose is doubled, over the range of doses used, and this relationship can be used

FIG. 5.—Dose-Response Graphs, Age Standardised. 128 Weeks. Upper: All Tumours.
Lower: Infiltrating Carcinomas

Neutral Fraction ——— ——— ———
Stored Condensate — — — — — — —
24 Hour Condensate ——————————
Doses measured in mg. F A S per application to relate the differences between condensates to the change in dose which would be needed to give equal responses, in the same way as in a toxicity bioassay.

Table IV shows the relative tumorigenicities calculated from the mean tumour-induction times and the proportion never developing tumours, and also the values calculated from the standardised rates. These latter values are more complicated because they contain the statistically significant anomaly in the stored condensate results (described fully in the Appendix) which was not apparent in the mean tumour-induction times.

The first important feature of this table is the uncertainty in the relative tumorigenicity even working with a total of 8000 mice. For example, the ratio for 24-hour condensate and neutral fraction at the 95% confidence limit, lies somewhere between 1·5 and 2·1. This illustrates strikingly how difficult quanti-

FIG. 6.—Time Response Graphs, All Tumours. Unstandardised.

Neutral Fraction ——— ——— ———
Stored Condensate – – – – – – – –
24 Hour Condensate —————————

tative work is in this field and it may be noted that had the experiment been carried out on only about 1/12 of this scale with 50 animals on each dose level, or 600 in the whole experiment, then the rates of tumorigenicity for 24-hour condensate and neutral fraction would have had confidence limits of at least 1·0 to 3·0 at the 95% level.

Within the limits used the tumorigenicity of each material increased linearly with the log dose so that even at the highest dose used there was no evidence that the point of saturation of the tumorigenic action was near. Not unexpectedly, the mean tumour-induction times for carcinomas and non-infiltrating carcinomas were longer than those for all tumours but it is of considerable interest that the relative tumorigenicities of 24-hour condensate, stored condensate and neutral

TABLE IV.—*Relative Tumorigenicities*

From all tumours, all carcinomas and infiltrating carcinomas combined.

| Dose levels | Denom. | From mean tumour-induction times and proportions of animals never with tumours: Neutral fract. | Stored cond. | 24-hour cond. | From standardised tumour rates at 128 weeks: Neutral fract. | Stored cond. | 24-hour cond. |
|---|---|---|---|---|---|---|---|
| 25, 50 | Neutral fract. | 1 | 1·10 | 1·77 | 1 | 0·89+ 20%−16% | 1·88+ 16%−13% |
| 100 | Neutral fract. | | | | 1 | 2·06+ 28%−22% | |
| 25, 50 | Stored cond. | .. | 1 | 1·59 | .. | 1 | 1·94%+ 20%−16% |
| 100 | Stored cond. | | | | .. | 1 | 1·06+ 26%−22% |

Symmetric conf. limits p = 0·95 are +15% − 11% of the ratio

The % figures are symmetric conf. limits p = 0·95

fraction were found to be in the same ratio using in turn each of these three tumour types, as can be seen from Table V. This suggests that the three condensates act in broadly the same way on the test animal and that the relative tumorigenicities which we have given do refer to intrinsic properties of these condensates.

TABLE V.—*Relative Tumorigenicity*

From mean tumour-induction times

| | All tumours | Carcinomas | Infiltrating carcinomas |
|---|---|---|---|
| Stored/Neutral | 1·07 | 1·08 | 1·15 |
| 24-hour/Neutral | 1·67 | 1·63 | 1·69 |
| 24-hour/Stored | 1·56 | 1·50 | 1·47 |
| 95% Conf. lim. (equal tails) | +29%−21% | +67%−36% | +75%−37% |

As stated earlier, it had often been surmised that non-volatile neutral components of cigarette smoke are responsible for the tumorigenicity of the smoke condensate, but until the present experiments it could only be considered as an inspired guess. Our results, using 95% one tail confidence limits, suggest that non-volatile neutral components account for something more than 50% of the tumorigenicity of 24-hour cigarette smoke condensate, and 80% of that of stored cigarette smoke condensate as defined in this report. It then follows that the compounds responsible for this effect are stable after collection for several weeks and are not affected by moderate heat treatment and chemical manipulation. It is unlikely that they are produced as artifacts in the process of making stored condensate.

The results certainly suggest that 24-hour condensate is more tumorigenic than stored condensate. While neither material contains what is usually termed the volatile fraction, there must be substances in 24-hour condensate which are lost or modified when evaporation is carried out to dryness; chemical analyses of these condensates are however not informative because at this stage we do not know to which of the large number of constituents attention should be directed. The results provide no evidence to decide whether any part of the activity of 24-hour condensate is due to its processing or storage.

In practical terms, an important feature of these results seems to be that in relation to mouse skin they show that there are stable non-volatile neutral carcinogens in cigarette smoke condensate which are worth serious attention and which in particular merit investigation by detailed fractionation.

### SUMMARY

The mouse-skin carcinogenicity of freshly prepared cigarette smoke condensate was compared at equivalent dose rates with that of a condensate which had been evaporated to constant weight on a boiling water bath and subsequently kept for several weeks, and with that of a neutral fraction of the condensate. The carcinogenicity in terms of several different response measures was linearly related to the logarithm of the dose, and the response to the three different materials was broadly similar, the freshly prepared condensate being more active than the other two materials used.

The results provide evidence that non-volatile neutral components of cigarette smoke contribute substantially to the mouse skin carcinogenicity of whole 24-hour old cigarette smoke condensate, as defined in this report and, for the first time, that the compounds responsible for this effect are stable from 24 hours after collection for several weeks, and that these compounds were not produced as artifacts in the processing leading to stored condensate. The substances responsible for the additional carcinogenicity of 24-hour old condensate have not been identified.

I should like to thank the scientists of member companies of the Tobacco Research Council for their advice and help in this experiment, and particularly Mr. W. S. Paige for suggesting and carrying out the statistical analyses in this report and for preparing the statistical Appendix. The experiment also owes much to Mr. T. Smith who was responsible for the preparation of the condensates, to Mr. D. V. D. Thorowgood who was responsible for the animal husbandry, and to those many junior technicians whose efforts have made this work possible.

### APPENDIX

### Statistical Considerations

W. S. PAIGE

Several techniques are described in the literature for the analysis of mouse tumour experiments. The earliest is due to Twort and Twort (1933) who used three methods. The first two depend upon comparisons of cumulative percentages of tumours with a standard curve, and the third estimates the time at which 25% of the animals would have developed tumours, in the absence of mortality, using a crude approximation to the tumour expectation of animals which died during the experiment. Irwin and Goodman (1946) compared these measures with the standard actuarial calculations, which were also used by Palmes, Orris and Nelson (1962) and Blanding *et al.* (1951) and recommended the use of expectation of tumourless life, when there is no mortality, as the most clearly interpretable measure. In our terms this is the mean tumour induction time. They considered that the date at which a particular tumour rate was reached was not in general a useful measure of response.

Bryan and Shimkin (1941) described the application of standard bioassay techniques to this problem, but they were concerned mainly with potent carcinogens and did not use efficient techniques for comparison in the presence of natural mortality. They also gave analyses of tumour-induction times (under the name " latent period ") showing that in some cases they were independent of dose. They advocated the use of the ED50, the dose which produced 50% response, for the comparison of different treatments; and suggested that the mean tumour induction time for this dose was a useful parameter. Their formula for the time at which an experiment could be safely terminated shows the necessity of continuing experiments with weak carcinogens until all the animals are dead. Lea (1945) criticised this paper by Bryan and Shimkin for its lack of emphasis on techniques for eliminating differences in mortality. He suggested, for experiments with continued or persistent treatments, a maximum likelihood procedure for estimating a lognormal distribution over time of the tumorigenic force, which made full allowance for mortality. This method, however, suffered from the disadvantage that it assumed that all animals would develop tumours if they lived indefinitely, which does not seem to be justified with control groups or weak carcinogens.

Blanding *et al.* (1951) described a technique in which an effective tumour rate is plotted on log probability paper to estimate the time at which the rate will be 50%. This effective rate is the number of animals which have borne tumours divided by the original number less deaths without tumours. This rate is neither an uncorrected rate nor a standardised rate, and it is difficult to use in any theoretical model of tumour incidence. They also recommend the use of a tumour potency the reciprocal of the time-to-50%-rate, but this does not seem nearly as suitable a measure as the equivalent dose considered below for use with large experiments on weak carcinogens, since it depends on the stability of the animal population used, and its variance depends upon the mean.

Wynder (for example, Wynder and Hoffmann, 1959) and other workers have used the number of tumours or the number of tumour-bearing animals at a particular date to compare treatments; but this method is open to objections, as described below, if the different treatments have different mortality rates. However, this approach was used as the starting point in an attempt to develop an improved analysis.

### ANALYSIS OF RATES

A number of different analyses have been done on our data, but they fall into two main types: the first deals with " tumour rates " defined as the number of tumour-bearing animals observed to date in a group divided by the number of animals in the group at the start of treatment. This is a cumulative measure which smooths out statistical fluctuations, but causes difficulties when independent statistical tests are needed for results at different stages of the experiment. The second group of analyses deals with the " tumorigenic force " defined as the number of new tumour-bearing animals observed in a group during a short time interval divided by the number of tumourless animals alive in the group at the start of that interval. The observations for different intervals are here uncorrelated, but suffer from large statistical fluctuations.

The simplest analysis which can be done is a comparison of the tumour rates at a given time for groups of animals under different treatments. In the case of toxic treatments (and 24-hour smoke condensate is somewhat toxic in the doses used in this experiment, since the mortality from all causes is higher under this treatment) there are two courses open. Firstly, the number of tumours which was actually observed can be used as it stands. If this is done a treatment which is toxic but has the same capacity for producing tumours as a non-toxic treatment will show a smaller number of tumours than the non-toxic treatment because the number of animals at risk at any time will be smaller if some have been poisoned. Alternatively, it can be assumed that the poisoned animals were a random sample of the whole. Then some form of correction can be applied for the varying mortalities to give standardised numbers of tumours and an analysis can be done of the response excluding the toxic effect. In order to do age standardisation by any technique it is necessary to assume that the expectation of life for those animals which develop tumours would, in the absence of treatment, be the same as that of the remainder of the group. This assumption is basic also in the analysis of tumorigenic force, since the denominators in all the fractions, for different treatments and for different times, must be comparable.

Detailed analyses of tumour rates and death rates were carried out at 24-week intervals from 56 weeks to 128 weeks from the start of treatment. Table II above shows, for all four rooms taken together, the average un-standardised rates at the four selected times, and Fig. 4 shows the results for 128 weeks graphically. Table III and Fig. 5 show corresponding age standardised rates. The age standardisation used the direct method (Yule, 1934), taking as its standard population the mean mortality curve for all animals in the experiment, which reduces the corrections needed almost to the minimum possible. For each week in turn the age-specific rate for a group of animals was applied to the proportion surviving of the standard population and these results were accumulated to give a standardised rate up to a given age for that group. To show the magnitude of these corrections the standardised death rates are also shown. In a group with greater than average mortality the standardised mortality will of course exceed 1 towards the end of the experiment. It was hoped originally to calculate mean tumour-induction period by the acturial method (Irwin and Goodman, 1946; Blanding *et al.*, 1951) but the observed rates were all well below 50% so the extrapolation involved made the results unreliable.

A preliminary analysis of deaths showed that as well as differences between the treatments there were several anomalies in the mortality curves for different animal rooms. This is shown in Table VI, which records the cumulative numbers of deaths. The most significant feature is the excess of deaths in Room 2 about week 70. This was due to animals being killed in Room 2 at that time on account of a number of different types of pathology, following a change in the standard of illness applied as a criterion for removal of animals. Similar but much smaller anomalies were found in other rooms at other stages in the experiment but no significant anomalies were found between groups of mice in a room. It was therefore necessary to keep the four animal rooms separate throughout the analysis (though the tables presented here give results for all four rooms together for brevity).

TABLE VI.—*Cumulative Number of Deaths (All Treatments)*

| Treatment Week | Room 1 | 2 | 3 | 4 |
|---|---|---|---|---|
| 56 | 512 | 537 | 531 | 551 |
| 72 | 944 | 1134 | 973 | 1000 |
| 88 | 1464 | 1589 | 1522 | 1501 |
| 104 | 1833 | 1890 | 1381 | 1851 |

Since there are anomalies in rates between the animal rooms, and because the corrections involved in standardising were quite large (as inspection of Table III shows) significance tests between treatments based upon binomial distribution theory, including the $\chi^2$ test, are invalid. Analysis of variance, however, does not depend upon the exact fit of the data to particular distributions, and is robust under deviation from distribution assumptions, so it can validly be applied to either the standardised or the unstandardised data.

### ANALYSIS OF VARIANCE

A preliminary inspection showed that the control groups, having few or no tumours, gave different variations between rooms from the treated groups. The analysis of variance was therefore split into two parts:

A *Controls* Two types of control (untreated and acetone treated ) $\times 4$ rooms.

B *Treated* 9 treatments $\times 4$ rooms. The 9 treatments were further divided into 3 types of condensate $\times$ 3 doses, the dose relationship being expressed as linear and quadratic terms. The between-rooms term, with 3 degrees of freedom, was subtracted from the residual as a " block effect ".

A specimen analysis of variance is shown in Table VII.

TABLE VII.—*Analysis of Variance* $Sin^{-1}$ *(SQRT (R))*

*R*=standardised rate, all tumours 104 weeks.

| Source | *df* | *SS* × 10⁴ | Mean square × 10⁴ | *F* |
|---|---|---|---|---|
| Total | 43 | 25055 | 583 | — |
| Between controls | 1 | 39·3 | 39·3 | 2·53 |
| Within controls | 6 | 93·1 | 15·5 | 1 |
| Control-treated | 1 | 9389·2 | 9389·2 | — |
| Dose linear | 1 | 10712 | 10712 | 436 |
| Dose quadratic | 1 | 87·3 | 87·3 | 3·55 |
| Condensates | 2 | 2312 | 1156 | 47·0 |
| Dose × cond. | 4 | 1064 | 266 | 10·8 |
| Within treat. | 24 | 591 | 24·6 | 1·00 |
| Rooms | 3 | 767 | 255·6 | 10·4 |

Analysis of variance cannot be done directly on the rates, since their variance depends upon the mean; the results must be transformed. The usual transformation for rates (Bartlett, 1947) is the inverse sine of the square root of the rate; but since ratios were required an analysis of the log rates was also done. The square root transformation appropriate for Poisson data was tried on some sets of results with no rates above 10%. These analyses showed little difference from the inverse sine results and were used only to confirm the findings.

The most striking feature of Table VII is the extreme linearity of the dose effect. In 24 analyses (4 times × 3 tumours × standardised or not) only four quadratic terms (for all tumours, 56 and 104 weeks) were significant at the $p = 0 \cdot 05$ level.

The other features of the analysis are shown best by the table of means, and that corresponding to Table VII is shown in Table VIII. As would be expected from Table VI, the difference between rooms is attributable to Room 2.

TABLE VIII.—*Means from Analysis of Variance Sin*$^{-1}$ (*SQRT* (*R*))

*R* = standardised rate, all tumours 104 weeks

| Controls: | Untreated | Acetone | C.L. |
|---|---|---|---|
| | 0·119 | 0·075 | 0·048 |

Treatments:

| Dose | Low | Med. | High | Mean |
|---|---|---|---|---|
| Neutral fraction | 0·248 | 0·396 | 0·548 | 0·397 |
| Stored condensate | 0·228 | 0·347 | 0·756 | 0·444 |
| 24-hour condensate | 0·350 | 0·618 | 0·789 | 0·586 |
| Mean | 0·275 | 0·454 | 0·698 | 0·476 |

C.L. individuals 0·049 row col. means 0·028
C.L. $p = 0 \cdot 95$

| Room | 1 | 2 | 3 | 4 | C.L. |
|---|---|---|---|---|---|
| | 0·442 | 0·555 | 0·461 | 0·445 | 0·032 |

C.L. $p = 0 \cdot 95$ denotes confidence limits at 95% level.

The interaction of Dose and Condensate is seen to be mainly due to the high dose treatment of stored condensate. The two lower doses show responses very similar to neutral fraction, while the high dose response is considerably higher than that for neutral fraction. This pattern is visible in all the analyses. It has been called the stored condensate anomaly.

There is a corresponding effect, in the opposite sense, in 24-hour condensate, where the top dose response is markedly low. This is not significant in many of the standardised analyses, but it is very significant in the unstandardised tables. It is thought that this is due to the toxic effect of large doses of 24-hour condensate.

Table IX summarises these effects in a number of analyses of variance in columns giving the dose effect, expressed as the increase in response for doubled dose, the differences between condensates, the difference between treated and control and the stored condensate anomaly expressed as difference observed—expected on linear increase. A few values are given for the log analysis, but this was little use for the carcinoma results since it is unsatisfactory with counts below about 5 individuals.

The great difficulty with the results in Table IX is to find some way of combining data for different times. It was clear from these results, however, that the response differences, on all the scales tried, varied with time, and it would be necessary to use the dose-response relationship to convert differences back into equivalent dose ratios.

TABLE IX.—*Analysis of Variance Estimates of Effects*

| Rate for | To week | Doubled Dose eff. | | Difference 24 hr-neut. | | Difference Stored-neut. | | Difference treat-control | | Stored anomaly (see p. 73) | | *F* Ratio for rooms (24, 3*df*) |
|---|---|---|---|---|---|---|---|---|---|---|---|---|
| | | Mean | C.L. | Mean | C.L. | Mean | C.L. | Mean | C.L. | Mean | C.L. | |
| | | | | | | Sin-1($SQRT(R)$) analysis × $10^2$ | | | | | | |
| Unstandardised | | | | | | | | | | | | |
| All tumours | 56 | 11·4 | 1·8 | 12·1 | 3·6 | 4·9 | 3·6 | 18·8 | 3·6 | 7·9 | 5·4 | 0·62 |
| | 80 | 13·6 | 1·4 | 14·9 | 2·7 | 4·4 | 2·7 | 28·0 | 3·1 | 8·2 | 4·0 | 6·30 |
| | 104 | 14·2 | 1·6 | 12·8 | 3·1 | 2·9 | 3·1 | 31·9 | 3·3 | 6·6 | 4·6 | 3·64 |
| | 128 | 13·6 | 1·8 | 11·4 | 3·6 | 1·7 | 3·6 | 32·4 | 4·0 | 6·6 | 5·4 | 1·53 |
| All carcinomas | 56 | 3·5 | 1·5 | 3·2 | 3·0 | 1·2 | 3·0 | 4·3 | 2·4 | 4·7 | 4·6 | 0·52 |
| | 80 | 8·3 | 1·6 | 7·4 | 3·2 | 4·8 | 3·2 | 14·8 | 2·4 | 5·6 | 4·8 | 0·26 |
| | 104 | 10·1 | 1·3 | 8·9 | 2·7 | 2·4 | 2·7 | 21·9 | 2·7 | 6·9 | 4·0 | 1·96 |
| | 128 | 9·7 | 1·2 | 9·7 | 2·5 | 3·6 | 2·5 | 23·5 | 2·6 | 6·3 | 3·7 | 2·63 |
| Infiltrating carcinomas | 56 | 2·1 | 1·4 | 3·0 | 2·8 | 1·8 | 2·8 | 3·2 | 2·3 | 4·5 | 4·2 | 1·08 |
| | 80 | 6·7 | 1·6 | 6·3 | 3·2 | 2·5 | 3·2 | 11·2 | 2·4 | 5·4 | 4·8 | 1·31 |
| | 104 | 8·1 | 1·5 | 6·8 | 3·0 | 2·4 | 3·0 | 16·6 | 2·3 | 6·8 | 4·5 | 1·17 |
| | 128 | 8·1 | 1·5 | 7·6 | 3·0 | 2·8 | 3·0 | 17·4 | 2·3 | 6·7 | 4·5 | 1·09 |
| Standardised | | | | | | | | | | | | |
| All tumours | 56 | 12·1 | 1·8 | 13·1 | 3·6 | 5·4 | 3·6 | 19·4 | 3·2 | 8·5 | 5·4 | 0·66 |
| | 80 | 17·1 | 1·5 | 18·0 | 3·1 | 5·4 | 3·1 | 30·4 | 3·3 | 10·6 | 4·6 | 11·58 |
| | 104 | 21·1 | 1·9 | 18·8 | 4·0 | 4·7 | 4·0 | 37·8 | 3·7 | 10·1 | 5·9 | 10·39 |
| | 128 | 21·0 | 1·9 | 19·1 | 3·8 | 5·0 | 3·8 | 38·4 | 3·8 | 10·5 | 4·7 | 9·80 |
| All carcinomas | 56 | 3·5 | 1·5 | 3·2 | 3·1 | 1·2 | 3·1 | 4·3 | 2·4 | 4·7 | 4·6 | 0·52 |
| | 80 | 9·0 | 1·7 | 7·6 | 3·5 | 5·1 | 3·5 | 15·2 | 2·4 | 6·1 | 5·2 | 0·35 |
| | 104 | 13·6 | 1·5 | 12·4 | 3·0 | 4·5 | 3·0 | 24·3 | 2·7 | 9·1 | 4·5 | 5·36 |
| | 128 | 14·7 | 1·7 | 14·8 | 3·4 | 5·3 | 3·4 | 25·8 | 2·8 | 8·8 | 5·1 | 4·55 |
| Infiltrating carcinomas | 56 | 2·1 | 1·4 | 3·1 | 2·8 | 1·8 | 2·8 | 3·1 | 2·3 | 4·5 | 4·2 | 1·08 |
| | 80 | 7·1 | 1·7 | 7·1 | 3·4 | 2·9 | 3·4 | 11·6 | 2·4 | 5·8 | 5·1 | 0·83 |
| | 104 | 10·5 | 1·8 | 8·2 | 3·6 | 4·7 | 3·6 | 18·7 | 2·5 | 9·0 | 5·3 | 2·12 |
| | 128 | 11·2 | 1·9 | 10·1 | 3·7 | 5·0 | 3·7 | 19·1 | 2·5 | 9·0 | 5·5 | 1·58 |
| | | | | | Log ($R$) analysis | | | | | | | |
| Unstandardised | | | | | | | | | | | | |
| All tumours | 80 | 0·81 | 0·10 | 0·85 | 0·20 | 0·15 | 0·20 | 3·75 | 1·28 | 0·40 | 0·30 | 4·6 |
| | 128 | 0·63 | 0·10 | 0·51 | 0·18 | −0·13 | 0·18 | 3·10 | 1·36 | 0·28 | 0·28 | 1·7 |
| Standardised | | | | | | | | | | | | |
| All tumours | 128 | 0·85 | 0·10 | 0·69 | 0·26 | 0·04 | 0·21 | 3·25 | 1·16 | 0·36 | 0·30 | 6·2 |

C.L. denotes half width of Confidence Limit P = 0·95 2 equal tails.

Since the linear relationship between the logarithm of dose and the response is then of great importance in interpretation of the results, an entirely different analysis, based upon the tumorigenic force, was done to check it.

### ANALYSIS OF TUMORIGENIC FORCE

Individual periods must be combined for this purpose in order to reduce the random variations, and a method of fitting standard statistical distributions to the tumorigenic force was used. This method is a generalisation of that used by Lea (1945) and approximated by Blanding *et al.* (1951) and it fits a lognormal distribution (with mean log tumour-induction time $m$, standard deviation $s$) to the tumorigenic force, using the method of maximum likelihood. The generalisation allows a proportion $c$ of the animals to remain tumourless indefinitely. This method was devised by Boag (1948, 1949) for examination of radiotherapy success in human cancer. Assuming that the distribution over time of the tumorigenic force in a given group of animals has either normal or lognormal form, it is then possible to write down the likelihood function for the observed data:

$$L = K + \sum_{\substack{\text{Group} \\ 1}} \ln \frac{(1 - c)\, z}{s} + \sum_{\substack{\text{Group} \\ 2}} \ln \left(c + (1 - c)\, q\right)$$

in which

$$x = \frac{t - m}{s} \quad \text{or} \quad \frac{\ln(t) - m}{s}$$

for the two alternative forms

$$z = \frac{1}{\sqrt{2\pi}}\, e^{-0 \cdot 5x^2} \quad \text{and} \quad q = \int_x^\infty z dx$$

where $L$ is the log likelihood, $t$ the time, $m$ the mean of the distribution, $s$ its standard deviation and $c$ the proportion of animals which would never develop tumours in the absence of natural mortality.

The summation over Group 1 covers all animals which develop tumours (tumour developing at time $t$) and that over Group 2 all animals which die tumourless (at time $t$).

The values of $m$, $s$, $c$ which maximise $L$ can be found either by equating partial differential coefficients to zero in the usual way (as was done by Boag) or by using a maximum seeking procedure such as Nelder's simplex method (Nelder and Mead, 1965). Computer program written in Algols are available for the latter technique.

The assumptions made about the form of the distribution of tumour incidence can then be rigorously tested by $\chi^2$ goodness of fit tests. The distribution parameters will contain the information about difference between treatments, and analyses can be done on them.

This model has two great advantages over methods using rates. The distribution of tumours over time is defined as a distribution of tumorigenic force, which makes it possible to eliminate from the equations the mortality of the various groups of animals, and get satisfactory comparisons even of toxic treatments. This is, of course, the object of age standardised rates, but the fitted model overcomes the great disadvantage of age standardisation: that in treatments with excess mortality a single tumour may, in the standardised results, be weighed up to 4 or 5 and in a treatment with less than normal mortality it may count only 0·2 or 0·3. The maximum likelihood equations give each observation the weight needed to maximise the precision of the estimate; no difficulties are encountered when comparing treatment responses with different treatment-associated mortality rates nor any difficulties associated with selecting arbitrary time segments in the experiments.

Goodness of fit tests were done on the lognormal distributions fitted, and the values of $\chi^2$ are shown in Table X. In Room 2 there are four significant values, but inspection of the data shows that all four are local anomalies between weeks 65 and 75, which are due to the change mentioned above in the standards of reporting illness and tumours. Since these results were so local it was not thought worthwhile to censor or attempt to smooth the data to eliminate them. One similar, though smaller anomaly was found in one treatment of Room 3. To give a more sensitive test of the fit of the model $\chi^2$ tests were done on the sum of all treatments in each animal room. These were all very significant but the general picture was the same, with large local anomalies. The deviations in the four animal rooms were quite different, and a test on all four rooms taken together though still significant, showed smaller individual $\chi^2$'s. As a further check that these anomalies were local, and not due to a badly fitting model, normal distributions of tumorigenic force were fitted: and both individual treatment $\chi^2$ tests and an overall $\chi^2$ test were done. The results of these tests were almost identical with those of the lognormal tests, helping to confirm the local variation hypothesis.

Analyses of the normal distributions were done, but it was found that the standard deviation of the distributions increased with the means; and the patterns of variation between doses and treatments were less clear than those shown by the lognormal distributions, so the normal distribution results are not given in detail.

TABLE X.—*Goodness of Fit Lognormal Model Tumours*

| Treatment | | Room 1 | | Room 2 | | Room 3 | | Room 4 | |
|---|---|---|---|---|---|---|---|---|---|
| No. | | *df* | $\chi^2$ | *df* | $\chi^2$ | *df* | $\chi^2$ | *df* | $\chi^2$ |
| 1 | Control | 1 | 0·02 | 2 | 0·92 | 1 | 0·25 | 2 | 0·80 |
| 2 | Control acet. | 1 | 0·00 | 1 | 0·38 | 1 | 0·06 | 1 | 0·35 |
| 3 | Neutral low | 3 | 2·48 | 3 | 2·33 | 2 | 0·67 | 4 | 1·10 |
| 4 | Neutral med. | 4 | 3·41 | 5 | 2·33 | 6 | 5·38 | 6 | 4·79 |
| 5 | Neutral high | 8 | 12·65 | 9 | 25·01 | 9 | 8·40 | 8 | 8·60 |
| 6 | Stored low | 1 | 1·01 | 3 | 1·79 | 3 | 4·28 | 2 | 0·10 |
| 7 | Stored med. | 6 | 7·32 | 7 | 14·51 | 4 | 2·98 | 4 | 1·57 |
| 8 | Stored high | 12 | 12·99 | 11 | 17·01 | 12 | 23·82 | 12 | 10·99 |
| 9 | 24-hour low | 5 | 1·28 | 7 | 4·66 | 6 | 5·88 | 4 | 1·00 |
| 10 | 24-hour med. | 11 | 18·18 | 11 | 35·19 | 9 | 9·23 | 8 | 4·74 |
| 11 | 24-hour high | 13 | 8·47 | 12 | 11·42 | 11 | 14·73 | 10 | 3·05 |

*Examples of Anomalies*

| Room 2 Treatment 10 | | | Room 3 Treatment 8 | | |
|---|---|---|---|---|---|
| Weeks | Obs. | Exp. | Weeks | Obs. | Exp. |
| 64–67 | 2 | 4·5 | 56–59 | 6 | 4·2 |
| 68–71 | 5 | 4·6 | 60–63 | 1 | 4·0 |
| 72–75 | 0 | 3·7 | 64–67 | 9 | 3·9 |
| 76–79 | 11 | 3·2 | 68–71 | 3 | 3·4 |
| 80–83 | 3 | 2·5 | — | — | — |

TABLE XI.—*Fitted Distributions—Means of Four Rooms-Lognormal Distributions*

| Treatment and dose level | Tumours | | | Carcinomas | | | Infiltrating carcinomas | | |
|---|---|---|---|---|---|---|---|---|---|
| | m | s | c | m | s | c | m | s | c |
| Controls, untreated | 5·362 | 0·517 | 0·438 | 5·745 | 0·304 | 0·607 | 5·975 | 0·332 | 0·744 |
| Controls, Acetone | 5·454 | 0·499 | 0·485 | 5·995 | 0·351 | 0·747 | 5·995 | 0·351 | 0·747 |
| Neutral fraction, low | 5·251 | 0·527 | 0·307 | 5·038 | 0·264 | 0·382 | 5·584 | 0·434 | 0·551 |
| Neutral fraction, med. | 4·664 | 0·309 | 0·226 | 4·896 | 0·284 | 0·319 | 5·211 | 0·385 | 0·408 |
| Neutral fraction, high | 4·530 | 0·345 | 0·165 | 4·729 | 0·272 | 0·259 | 4·822 | 0·300 | 0·289 |
| Stored condensate, low | 4·952 | 0·383 | 0·316 | 5·121 | 0·277 | 0·411 | 5·594 | 0·337 | 0·587 |
| Stored condensate, med. | 4·766 | 0·397 | 0·240 | 4·895 | 0·295 | 0·326 | 5·157 | 0·388 | 0·401 |
| Stored condensate, high | 4·409 | 0·462 | 0·089 | 4·575 | 0·264 | 0·196 | 4·647 | 0·272 | 0·223 |
| 24 hr. condensate, low | 4·788 | 0·445 | 0·240 | 4·968 | 0·353 | 0·327 | 5·228 | 0·491 | 0·390 |
| 24 hr. condensate, med. | 4·490 | 0·397 | 0·139 | 4·660 | 0·257 | 0·237 | 4·831 | 0·325 | 0·280 |
| 24 hr. condensate, high | 4·396 | 0·444 | 0·086 | 4·607 | 0·231 | 0·217 | 4·731 | 0·263 | 0·269 |

m = mean tumour induction time.

s = standard deviation of tumour induction time.

c = proportion which would never develop tumours.

m and s are expressed in terms of $\log_e$ (weeks) and c in terms of proportion.

Table XI gives the means over four rooms of the three lognormal distribution parameters. The same analysis of variance as for rates, but without transformation, was done on the three parameters. It was found that the standard deviation *s* did not significantly change from one treatment to another or between dose levels. In Tables XII and XIII have been extracted the mean tumour-induction times for the three dose rates and for the three treatments.

TABLE XII.—*Means of Lognormal Distributions*

| | Tumours | | Carcinomas | | Infiltrating carcinomas | |
|---|---|---|---|---|---|---|
| | log | weeks | log | weeks | log | weeks |
| Low dose | 4·913 | 136 | 5·042 | 155 | 5·469 | 238 |
| Medium dose | 4·640 | 103 | 4·817 | 124 | 5·066 | 159 |
| High dose | 4·445 | 85 | 4·637 | 102 | 4·734 | 114 |
| C.L. ($p = 0·95$) | 0·053 | — | 0·090 | — | 0·176 | — |
| $\chi^2$ dose effect | 0·234 | — | 0·203 | — | 0·367 | — |
| C.L. ($p = 0·95$) | 0·038 | — | 0·064 | — | 0·124 | — |

TABLE XIII.—*Means of Lognormal Distributions*

| | Tumours | | Carcinomas | | Infiltrating carcinomas | |
|---|---|---|---|---|---|---|
| | log | weeks | log | weeks | log | weeks |
| Neutral fraction | 4·731 | 114 | 4·888 | 137 | 5·206 | 156 |
| Stored condensate | 4·709 | 110 | 4·863 | 135 | 5·132 | 152 |
| 24-hr. condensate | 4·558 | 95 | 4·745 | 104 | 4·930 | 139 |
| C.L. ($p = 0·95$) | 0·053 | — | 0·090 | — | 0·176 | — |
| Mean | 4·666 | — | 4·832 | — | 5·089 | — |

The fall of the mean tumour-induction time as the dose increases is not significantly different from a linear change (in the log time); and the differences in the rate of change between the three tumour responses are not significant, the average fall for a doubled dose being 0·235 ± 0·031 or about 27%.

The three types of condensate show the same pattern in all three responses with stored condensate having a mean a little below that of neutral fraction (0·024 or 3%) and 24-hour old condensate appreciably lower (0·177 or 19%). The means differ significantly for the three tumour responses in the order all tumours (lowest), carcinomas, and infiltrating carcinomas (highest). The stored condensate anomaly and the similarity of 24-hour condensate high and 24-hour condensate medium dose rates can still be seen but they are not significant. For the proportion of animals which would never develop tumours (*c*) the difference between treatments are consistent, showing a fall of about 1% between neutral fraction and stored condensate and about 7% between neutral fraction and 24-hour condensate. The stored condensate anomaly is significant only in the analysis of *c* for all tumours.

### MEASUREMENT OF TUMORIGENICITY

There are many response scales which can be used to measure tumorigenicity for example tumour rates at different times in the experiment. These scales will give different ratios for the relative response of two substances as shown in Table

IX. In many cases however the response ratio for two substances varies from scale in the same way as the response to different doses of a single substance. A dose level of one substance can be found which would give the same response in every scale as a known dose of a second substance. Relative tumorigenicity can then be defined as a dose ratio rather than a response ratio, in a way analogous to LD 50 in toxicity trials. Toxicity trials are however simpler than carcinogen assays because they usually last a relatively short time, while the carcinogen assays must cover nearly the whole life span of the test animals, and it must be shown that a relative tumorigenicity does not change with time during the trial.

To fulfil this requirement the response-time curves for different substances must have the same shape (though they may vary both in magnitude of response and in time of appearance of tumours). The condition for making valid comparisons of tumorigenicity may be defined:

> The relative tumorigenicity of two substances is $r$ if, and only if, a dose $D$ of one produces the same distribution of tumour incidence as a dose $rD$ of the second, when these doses are administered in the same way to random samples of a single population of animals.

This definition implies that in many cases it will not be possible to define a relative tumorigenicity because the shapes of distributions of incidence are not sufficiently similar.

In the present work this type of comparison was justified by showing that the time-response curves for individual treatments all fitted the lognormal distributions, so that all the information could be concentrated into the three parameters $m$, $s$ and $c$, which proved to be highly correlated over the population of 44 groups of mice and three responses. Either $m$ or $c$ could therefore be considered as a single response parameter permitting a valid measurement of " relative tumorigenicity " to be made for the three treatments.

As a check on the fit of this model, graphs of the tumorigenic force $T$ were prepared. These all showed that $T$ increased with time; but when straight lines were fitted, allowing for the decreasing weight of the observations as the number of animals falls, it was found that the slopes were proportional to the mean, so that the condition was fulfilled. The similarity is shown by Fig. 7, in which the uncorrected number of tumour-bearing animals for neutral fraction high dose has been matched by interpolating between 24-hour condensate medium and low doses.

The analysis of variance of the tumour-induction time $m$ or proportion of animals which never develop tumours $c$ can then be used to interpolate between the responses for different dose levels to get relative carcinogenicities for different smoke condensates and Fisher's method can be used to obtain fiducial limits for the ratios (Fisher, 1946). No difference was found between the results (Table V above) obtained with either parameter or those for all tumours, carcinomas and infiltrating carcinomas and the mean ratios of carcinogenicity are given in Table IV. This similarity of the curves obtained for the different condensates and types of tumour suggests that the mechanism of tumour production is the same for all three types of condensate.

The use of ratios obtained using the lognormal model depends of course on the assumptions:

(a) The model is realistic and since the tests of fit are not unsatisfactory the parameters obtained do in fact represent the data.

FIG. 7.—Similarity of shape for Time Response curves. The circles show the Neutral Fraction High Dose results for one room. The solid line is a linear interpolation between 24 hour condensate medium and low curves.

(b) The division of information shown by the analysis of variance, with dose response relationship in $m$ and $c$ and random variations in $s$ has not in fact destroyed relevant information on dose response relationships.

If either of these assumptions has to be rejected the results must be expressed using incidence rate measures of response, as in the right-hand side of Table IV.

### CONCLUSIONS

Two important conclusions of interest to statisticians can be drawn from this experiment.

1. In tests of weak carcinogens where it is essential to use the maximum possible dose, and to continue treatment until death, there will often be differences in mortality between dose levels and treatments. It is important to allow for this in the analysis of the results, if it is possible to make the basic assumptions so that correction is possible.
2. The lognormal distribution of tumorigenic force, with some animals never developing tumours, fits the data from this experiment. It provides a simpler picture than the other analyses which have been done.

### REFERENCES

BARTLETT, M. S.—(1947) *Biometrics*, **3**, 39.

BENTLEY, H. R. AND BURGAN, J. G.—(1961) ‘ Cigarette smoke condensate : preparation and routine laboratory estimation ’. 2nd edition. London (Tobacco Manufacturers’ Standing Committee) Research Paper No. 4.

BLANDING, F. H., KING, W. H. JR., PRIESTLEY, W. JR., AND REHNER, J. JR.—(1951) *Archs ind. Hyg.*, **4**, 335.
BOAG, J. W.—(1948) *Br. J. Radiol.*, **21**, 128 and 189.—(1949) *Jl R. statist. Soc.*, B., **11**, 15.
BRYAN, W. R. AND SHIMKIN, N. B.—(1941) *J. natn. Cancer Inst.*, **1**, 807.
DAY, T. D.—(1961) *Rep. Br. Emp. Cancer Campn*, **39**, 452.
FISHER, R. A.—(1946) 'Statistical Methods for Research Workers' 10th edition. Edinburgh (Oliver & Boyd) p. 142.
IRWIN, J. O. AND GOODMAN, N.—(1946) *J. Hyg., Camb.*, **44**, 362.
LAURENE, A. H. AND HARRELL, T. G.—(1958) *Analyt. Chem.*, **30**, 1800.
LEA, D. E.—(1945) *Cancer Res.*, **5**, 633.
NELDER, J. A. AND MEAD, R.—(1965) *Comput. J.*, **7**, 308.
PALMES, E. D., ORRIS, L. AND NELSON, N.—(1962) *Am. ind. Hyg. Ass. J.*, **23**, 257.
TWORT, C. C. AND TWORT, J. M.—(1933) *Am. J. Cancer*, **17**, 293.
WILLITS, C. O., SWAIN, M. L. AND CONNELLY, J. A.—(1950) *Analyt. Chem.*, **22**, 430.
WYNDER, E. L., GRAHAM, E. A. AND CRONINGER, A. B.—(1955) *Cancer Res.*, **15**, 445.
WYNDER, E. L. AND HOFFMANN, D.—(1959) *Cancer, N.Y.*, **12**, 1079. (1964) *Adv. Cancer Res.*, **8**, 250.
YULE, G. U.—(1934) *Jl R. statist. Soc.*, **97**, 1.